# Integrating HECW1 expression into the clinical indicators exhibits high accuracy in assessing the prognosis of patients with clear cell renal cell carcinoma

**DOI:** 10.1186/s12885-021-08631-9

**Published:** 2021-08-04

**Authors:** Chao Wang, Keqin Dong, Yuning Wang, Guang Peng, Xu Song, Yongwei Yu, Pei Shen, Xingang Cui

**Affiliations:** 1 Department of Urinary Surgery, Gongli Hospital, Second Military Medical University (Naval Medical University), 219 Miaopu Road, Shanghai, China; 2 Department of Urology, the Affiliated Changzhou No. 2 People’s Hospital of Nanjing Medical University, 29 Xinglong Road, Changzhou, Jiangsu China; 3 Department of Urinary Surgery, The Third Affiliated Hospital of Second Military Medical University (Eastern Hepatobiliary Surgery Hospital), 700 North Moyu Road, Shanghai, China; 4 Department of Orthopedic, Joint Logistic Support Force NO.925 Hospital of PLA, 67 Yellow River Road, Guiyang, 550009 Guizhou China; 5 Department of Urinary Surgery, Joint Logistic Support Force NO.925 Hospital of PLA, 67 Yellow River Road, Guiyang, 550009 Guizhou China; 6 Department of Urology, the Seventh People’s Hospital Affiliated to Shanghai University of Traditional Chinese Medicine, 358 Datong Road, Pudong New Area, Shanghai, China; 7 Department of Pathology, Changhai Hospital, Second Military Medical University (Naval Medical University), 168 Changhai Road, Shanghai, China; 8 Department of Nephrology, Gongli Hospital, Second Military Medical University (Naval Medical University), 219 Miaopu Road, Shanghai, China

**Keywords:** Clear cell renal cell carcinoma, HECW1, Prognostic marker, Disease progression

## Abstract

**Background:**

Although many intratumoral biomarkers have been reported to predict clear cell renal cell carcinoma (ccRCC) patient prognosis, combining intratumoral and clinical indicators could predict ccRCC prognosis more accurately than any of these markers alone. This study mainly examined the prognostic value of HECT, C2 and WW domain-containing E3 ubiquitin protein ligase 1 (HECW1) expression in ccRCC patients in combination with established clinical indicators.

**Methods:**

The expression level of HECW1 was screened out by data-independent acquisition mass spectrometry (DIA-MS) and analyzed in ccRCC patients from the The Cancer Genome Atlas (TCGA) database and our cohort. A total of 300 ccRCC patients were stochastically divided into a training cohort and a validation cohort, and real-time PCR, immunohistochemistry (IHC) and statistical analyses were employed to examine the prognostic value of HECW1 in ccRCC patients.

**Results:**

The expression level of HECW1 usually decreased in human ccRCC specimens relative to control specimens in TCGA (*p* < 0.001). DIA-MS, Real-time PCR, and IHC analyses also showed that the majority of ccRCCs harbored decreased HECW1 expression compared with that in normal adjacent tissues (*p* < 0.001). Additionally, HECW1 expression was reduced in ccRCC cell lines compared with the normal renal cell line HK-2 (*p* < 0.001). Moreover, lower HECW1 expression was found in ccRCC patients with a higher tumor node metastasis (TNM) stage, bone metastasis, or first-line targeted drug resistance (*p* < 0.001). Low HECW1 expression indicated higher TNM stage, SSIGN (Stage, Size, Grade, and Necrosis) score and WHO/ISUP grade and poor prognosis in ccRCC patients (*p* < 0.05). Even after multivariable adjustment, HECW1, TNM stage, and SSIGN score served as independent risk factors. The c-index analysis showed that integrating intratumoral HECW1 expression into TNM stage or SSIGN score resulted in a higher c-index value than these indicators alone for predicting ccRCC patient prognosis.

**Conclusion:**

HECW1 is a novel prognostic biomarker and therapeutic target in ccRCC, and integrating intratumoral HECW1 expression with established clinical indicators yields higher accuracy in assessing the postoperative prognosis of ccRCC patients.

**Supplementary Information:**

The online version contains supplementary material available at 10.1186/s12885-021-08631-9.

## Background

Clear cell renal cell carcinoma (ccRCC) is the most common type of kidney cancer, which ranks as the 6th and 9th most frequent cancers in men and women, respectively, in the United States [1]. Although radical nephrectomy, targeted therapies and immunotherapy have been developed for ccRCC, recurrence and metastasis still exist, which results in a poor prognosis for ccRCC patients [2]. Therefore, it is important to search for reliable prognostic biomarkers to monitor postoperative disease progression and recurrence, which is critical for clinical decision making [3].

In the clinic, tumor node metastasis (TNM) staging has been applied to evaluate the outcome of ccRCC patients [4]. The stage, size, grade and necrosis (SSIGN) scoring system and the University of California Los Angeles Integrated Staging System (UISS) stratify ccRCC patients into low-risk, intermediate-risk, and high-risk prognostic groups [4]. In addition, many molecular prognostic indicators for ccRCC have been reported, such as BAP1, PBRM1, PDZK1, and CBX4 [4–6]. However, there are still some limitations to these indicators, which cannot completely and accurately evaluate the prognosis of ccRCC patients.

Recently, our research group has employed data-independent acquisition mass spectrometry (DIA-MS) on 230 ccRCC patients and screen out the proteins related to ccRCC patients’ prognosis. Among the proteins, we found that HECT, C2 and WW domain containing E3 ubiquitin protein ligase 1 (HECW1) was significantly down-regulated in ccRCC. HECW1, also named NEDL1, belongs to the E3 ligase HECT family [7]. HECW1 expression was initially identified in neuronal tissues, including the spinal cord [8]. HECW1 binds to the COOH-terminal region of p53, which promotes its transcriptional activation and proapoptotic function [9]. Furthermore, HECW1 has been described in some studies on malignant tumors. Exome sequencing studies have revealed somatic mutations in HECW1, along with other novel driver genes, in non-small cell lung cancer (NSCLC) [10]. In addition, the novel mutant gene HECW1 was identified in muscle-invasive transitional cell carcinoma [11]. Moreover, HECW1 has been shown to degrade thyroid transcription factor 1 in follicular thyroid carcinoma cells [12]. However, the expression and prognostic significance of HECW1 in ccRCC is unknown.

In the present research, The Cancer Genome Atlas (TCGA) database and two cohorts of ccRCC patients were employed to detect HECW1 expression in ccRCC and to determine whether HECW1 expression is associated with disease progression and postoperative prognosis in ccRCC patients. Furthermore, the prognostic accuracy of HECW1, TNM stage, and SSING score were compared and integrated to achieve a reliable prognostic model.

## Methods

### Collection and analysis of public databases

Datasets from The Cancer Genome Atlas (TCGA) were downloaded from National Cancer Institute GDC Data Portal (https://portal.gdc.cancer.gov) using FirebrowseR package. The KIRC dataset from all TCGA cohorts were selected and 508 samples from ccRCC patients were filtered out by barcodes and gene mRNA expression profiles. Then, the gene expression profiles were normalized by using DESeq2 package. The expression of HECW1 was compared between 508 tumor samples and 72 normal adjacent tissues, and then also compared in 69 paired samples. Wilcox test was used to compare the expression of HECW1 between the ccRCC and normal adjacent tissues.

### Data-independent acquisition mass spectrometry (DIA-MS)

Formalin-fixed paraffin embedding (FFPE) tissue blocks of 230 ccRCC patients were organized by seasoned biopsy pathology doctors. A perforated sampler was used to drill into the FFPE tissues blocks and extract tissue cores (1 mm in diameter and about 1–1.5 mg in weight). Three tissue cores were made for each case as biological repeat samples. The DIA MS acquisition of peptides was performed on Dionex Ultimate 3000 RSLC Nano System.

### Patients and specimens

300 ccRCC patients, who were pathologically diagnosed between 2012 and 2014 from Eastern Hepatobiliary Surgery Hospital (Shanghai, China), were recruited in this study to determine the prognostic significance of HECW1. The type of ccRCC tissues used in our study are postoperative ccRCC specimens. 58% (174/300) patients received partial nephrectomy while others (126/300) underwent radical nephrectomy. Another 60 paired ccRCC specimens were used for Real-time PCR analysis. This study followed the recommendations for prognostic studies of tumor biomarkers (REMARK) [13]. All experiments were approved by the institutional ethical review boards from all hospitals, and all written informed consents were obtained from the ccRCC patients. The clinical features of the ccRCC patients are listed in Table 1. Pathologic specimens were evaluated by two surgical pathologists, with stage and grade determined according to the 2017 American Joint Committee on Cancer guidelines and WHO/ISUP grade, respectively.

**Table 1:** Demographics and characteristics of patients with clear cell renal cell carcinoma (ccRCC)

Characteristics	NO. of cases(%)				Sum(***n =*** 300)
	training cohort(***n*** = 180)	validation cohort(***n*** = 120)	training cohort(***n =*** 150)	validation cohort(***n =*** 150)	
**Age(16y ~ 84y)**					
< 60	110(61.1)	72(60)	81(54)	101(67.3)	182(60.7)
≥ 60	70(38.9)	48(40)	69(46)	49(32.7)	18(39.3)
**Gender**					
Male	127(70.6)	88(73.3)	110(73.3)	105(70)	215(71.7)
Female	53(29.4)	32(26.7)	40(26.7)	45(30)	85(28.3)
**WHO/ISUP Grading**					
I-II	137(76.1)	91(75.8)	108(72)	120(80)	228(76)
III-IV	43(23.9)	29(24.2)	42(28)	30(20)	72(24)
**TNM stage**					
I-II	161(89.4)	105(87.5)	136(90.7)	130(86.7)	266(88.7)
III-IV	19(10.6)	15(12.5)	14(9.3)	20(13.3)	34(11.3)
**SSIGN**					
1–4	167(92.8)	114(95)	139(92.7)	142(94.7)	281(93.7)
≥ 5	13(7.2)	6(5)	11(7.3)	8(5.3)	19(6.3)

*SSIGN* Stage, Size, Grade, and Necrosis, *TNM* Tumor Node Metastasis

### Real-time polymerase chain reaction (real-time PCR)

The Real-time PCR assay was described as we reported previously [14]. Briefly, total RNAs were extracted with RNAiso Plus (9108, Takara, Kusatsu, Japan) and their corresponding cDNAs were synthesized using a PrimeScript One Step RT reagent Kit (RR037A, Takara, Kusatsu, Japan). Real-time PCR was taken with SYBR Green Real-Time PCR Master Mix (QPK201, Toyobo, Osaka, Japan) on an ABI PRISM 7300HT Sequence Detection System (Thermo Fisher Scientific, Waltham, USA). The primer sequences are shown: *HECW1* (forward primer, 5`- ACTGCTGCTGGAAGACGGTGAA-3`, and reverse primer, 5`- TCCTCCTCCTGCTCCTTCTCCT-3`), *GAPDH* (forward primer, 5′-GGAAGGTGAAGGTCGGAGT-3′, and reverse primer, 5′-CCTGGAAGATGGTGATGGG-3′). All results were normalized to the expression of GAPDH and fold change relative to the mean value was determined by 2^-△△Ct^.

### Immunohistochemistry (IHC)

The ccRCC pathological sections were dewaxed, hydrated, and repaired with citric acid buffer (1:100) in a pressure cooker (3 min) before cooling to room temperature. The pathological sections were then incubated with the reagents of the hypersensitive immunohistochemical kit (Fuzhou Maixin Biological Company, Fuzhou, China). The endogenous peroxidase blocker (within kit) was incubated for 30 min, and the animal non-immune serum blocker (within kit) was incubated for 20 min. Then rabbit anti-HECW1 antibody (ab121264, abcam, Cambridge, USA) or IgG antibody (ab37415, abcam, Cambridge, USA) was incubated at 4 °C overnight. The next day, the antibody was recovered and then incubated for 30 min with biotin labeled secondary antibody reagent (within kit), and then incubated with Streptomycetes antibiotic protein peroxidase reagent (within kit) for 30 min. Before and after each step of the above experiment, wash with PBS buffer for 3 times (5 min/time). Then, DAB color development kit (Fuzhou Maixin Biological Company, Fuzhou, China) was used for staining (terminating according to different antibody reaction time). The nucleus was stained with hematoxylan for 5 min, followed by differentiation solution for 2 s, blue return solution for 3 min, and finally transparent and sealed slices were performed. The IgG, positive, and negative controls for HECW1 have presented in supplementary Fig. S1F-G.

The presence of IHC staining for HECW1 was scored semiquantitatively as negative (0), weakly positive (1+), moderately positive (2+), or strongly positive (3+), and the percentages of positive cells were also determined. For each observed tissue component, a summary value referred to as component H-Score was calculated by the multiplication of the intensity score, which ranged from 0 to 3, by the percentage of positive cells, which ranged from 0 to 100, and the total H-Score for a tissue section was derived as the sum of the component H-Scores weighted by the fraction of each component observed in the tissue section (Supplementary Table S1).

### Cell culture

The ccRCC cell lines were bought from the Cell Bank of the Type Culture Collection of the Chinese Academy of Sciences (Shanghai, China) in 2019. HK-2 cells were cultured in high-glucose Dulbecco’s Modified Eagle’s Medium (DMEM) (12,100,046, Gibco, Waltham, USA). 786-O and 769-P cells were cultured in RPMI-1640 medium (C11875500BT, Gibco, Waltham, USA). Caki-1 cells were culture in McCoy’s 5A Medium (16600–082, Gibco, Waltham, USA). L-glutamine is contained in the culture medium. The culture medium of all cell lines were supplemented with fetal bovine serum (FBS, 10%, 16,140,071, Gibco, Waltham, USA) and 1% penicillin/streptomycin (15140–122, Gibco,Waltham, USA), and the FBS was heat-inactivated (56 °C, 30 min) before using. The cell lines were cultured at 37 °C in 5% CO_2_. Sunitinib and pazopanib-resistant 786-O cell lines (786-O-SR and 786-O-PR) were established as described in our previous research [14, 15]. The cell lines in this study were authenticated by short tandem repeat (STR) profiling and detected for mycoplasma contamination using a Mycoplasma Detection Kit (Biotool, Neuhof, Switzerland), and the most recent tests were conducted in October 2020. All cell lines used in the study were cultured within 40 passages.

### Statistical analysis

Numerical data were expressed as the mean ± S.D. Two-tailed Student’s t-test or Wilcoxon test was conducted for continuous variables. Chi-square test or fisher’s exact test was conducted for categorical variables. Time-dependent receiver operating characteristic (ROC) analysis was performed using ‘survivalROC’ package to determine the optimal cut-off values of the H-scores of HECW1. Survival curves were plotted using Kaplan-Meier analysis and compared via log-rank test. Variables with *p* values < 0.05 in univariate Cox proportional hazards analysis were included in multivariate analysis. Difference was considered significant at *p* < 0.05. Prognostic accuracy of the HECW1 classifier and other prognostic indicators was indicated by Harrell’s concordance index using ‘rms’ package(c-index). All the statistical analyses were performed using R-software (version 3.5.2).

## Results

### HECW1 expression is decreased in clear cell renal cell carcinoma

**Fig. 1:**
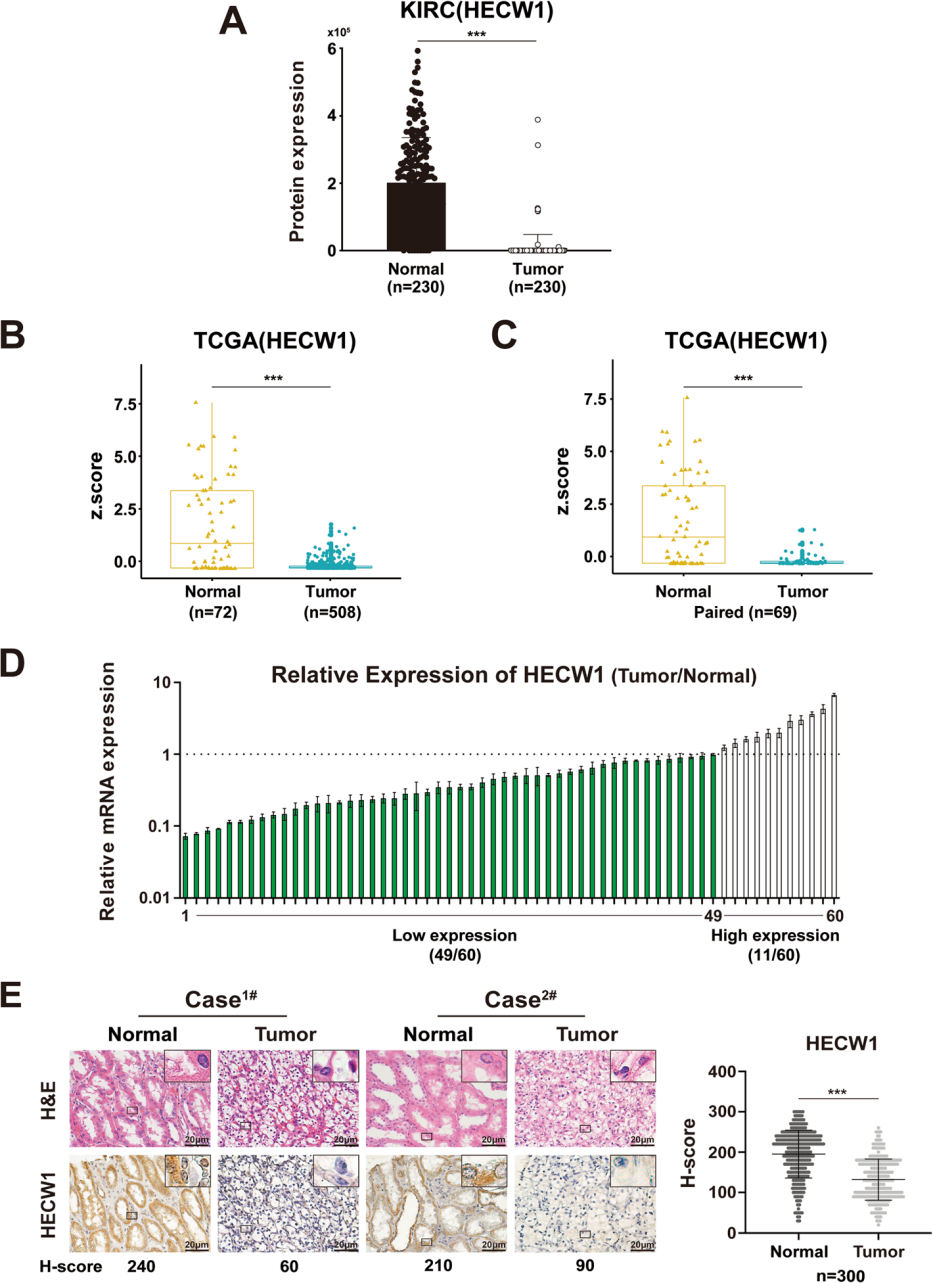
HECW1 expression is decreased in clear cell renal cell carcinoma. (**a**), The protein expression of HECW1 in ccRCC samples was determined by Data-independent acquisition mass spectrometry (DIA-MS) assay. (**b**) The expression of HECW1 in ccRCC tissues (Tumor, *n* = 508) and normal adjacent tissues (Normal, *n =* 72) from The Cancer Genome Atlas (TCGA) datasets was analyzed. The normalized read counts are shown in boxplots to compare the expression differences. (**c**) The expression of HECW1 between ccRCC tumor tissues (*n =* 69) and normal adjacent tissues (*n =* 69) from TCGA datasets was analyzed the same way as described above. (**d**) Real-time PCR was used to detect the relative mRNA expression of HECW1 in tumor samples and their paired normal adjacent tissues from ccRCC patients (*n* = 60). (**e**) Representative images of hematoxylin and eosin (H&E) staining and immunohistochemistry (IHC) staining for HECW1 in ccRCC tissues and normal adjacent tissues are presented (scale bar = 20 μm). The expression of HECW1 evaluated by the H-score method (for details, see *Materials and Methods*) in corresponding tissues is shown, and the values are represented as the mean ± SD (***p < 0.001; Wilcoxon test)

The protein expression of HECW1 in ccRCC samples was first determined by data-independent acquisition mass spectrometry (DIA-MS) assay, which showed that HECW1 expression was reduced in ccRCC (Fig. 1a). The expression of HECW1 was then analyzed in unpaired or paired ccRCC and normal adjacent tissues from the TCGA. Lower HECW1 expression was found in ccRCC in contrast to normal adjacent samples (Fig. 1b-c). To validate this initial finding, matched postoperative ccRCC specimens and their normal adjacent tissues were employed. As expected, the expression level of HECW1 was down-regulated in ccRCC in contrast to the adjacent renal tissues (Fig. 1d). Moreover, immunohistochemistry (IHC) assays were performed with postoperative ccRCC specimens and demonstrated that the majority of ccRCCs harbored decreased HECW1 expression compared with that in paired normal adjacent tissues (Fig. 1e). These findings indicate that the expression of HECW1 is commonly reduced in ccRCC.

### Low HECW1 expression indicates the progression of ccRCC

**Fig. 2:**
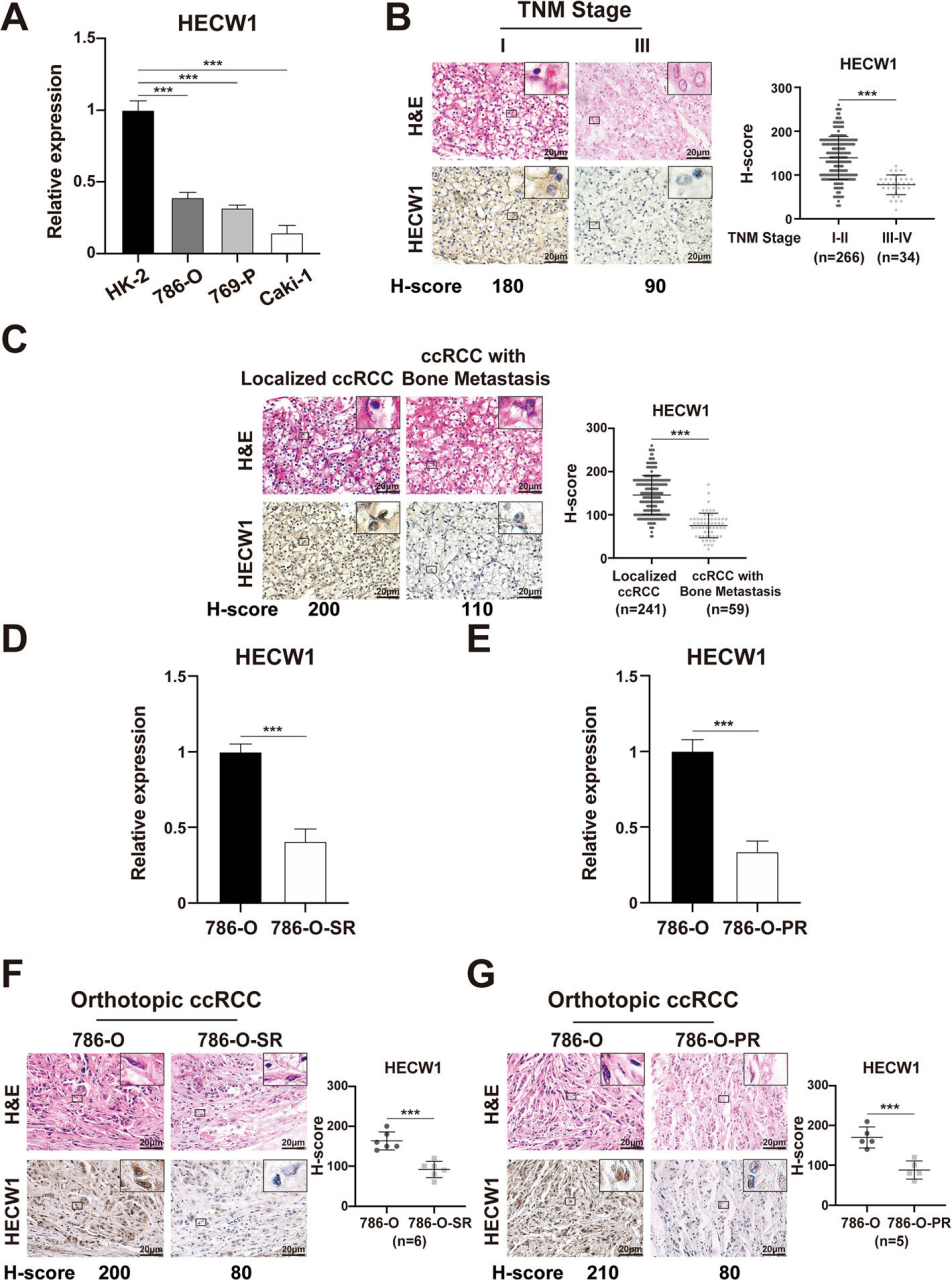
HECW1 expression is negatively associated with tumor stage, bone metastasis, and targeted drug resistance in ccRCC. (**a**) Real-time PCR was used to detect the mRNA expression of HECW1 in HK-2, 786-O or 769-P, and Caki-1 cells. (**b**) Representative images of H&E and IHC staining for HECW1 in ccRCC tissues with different tumor node metastasis (TNM) stages are shown (scale bar = 20 μm). The expression of HECW1 in ccRCC specimens was evaluated by the H-score method, and the values are represented as the mean ± SD. (**c**) Representative images of H&E and IHC staining for HECW1 in tissues of localized ccRCC and ccRCC with bone metastasis are shown (scale bar = 20 μm). The expression of HECW1 in ccRCC specimens was evaluated by the H-score method, and values are represented as the mean ± SD. (**d-e**) Real-time PCR was employed to detect the mRNA expression of HECW1 in 786-O-SR (**d**) and 786-O-PR (**e**) cells. (**f-g**) Representative images of H&E and IHC staining for HECW1 in sunitinib-resistant (**f**) or pazopanib-resistant (**g**) orthotopic ccRCC specimens are presented (scale bar = 20 μm). Values are represented as the mean ± SD. All *p* values are defined as ***p < 0.001

As the expression level of HECW1 is usually decreased in ccRCC, we postulated that HECW1 expression might be negatively associated with malignant characteristics of ccRCC. First, a real-time PCR assay was employed to show that the expression of HECW1 was reduced in the ccRCC cell lines 786-O, 769-P, and Caki-1 compared with that in the normal renal cell line HK-2 (Fig. 2a). Additionally, IHC assays presented that down-regulated HECW1 expression was observed in ccRCC specimens with a high TNM stage in contrast to that with a low TNM stage (Fig. 2b). Moreover, HECW1 expression was reduced in ccRCC specimens with bone metastasis compared with those without metastasis (Fig. 2c). Furthermore, the mRNA expression of HECW1 was down-regulated in sunitinib- or pazopanib-resistant 786-O cells (786-O-SR or 786-O-PR, as we described previously [14, 15] in contrast to that in naïve 786-O cells (Fig. 2d-e). Additionally, IHC assays demonstrated that downregulation of HECW1 was observed in sunitinib- or pazopanib-resistant orthotopic ccRCC specimens (which were also described in our previous studies [14, 15] compared with naïve orthotopic ccRCC specimens (Fig. 2f-g). These results exhibit that low HECW1 expression indicates higher tumor stage, bone metastasis, and targeted drug resistance of ccRCC.

### Low HECW1 expression is predictive of unfavorable prognosis in ccRCC patients

**Fig. 3:**
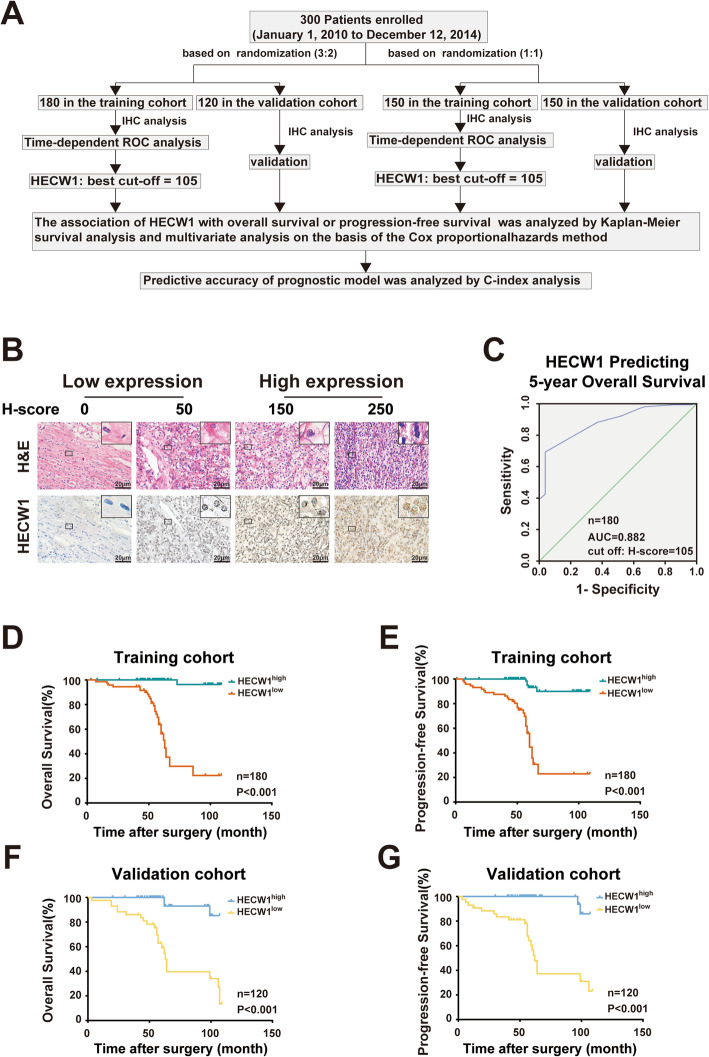
Low HECW1 expression is predictive of unfavorable clinicopathological characteristics and poor postoperative prognosis in ccRCC patients. (**a**) A flow chart of the present study is shown. (**b**) Representative images of H&E and IHC staining of HECW1 in ccRCC specimens are presented (scale bar = 20 μm). The expression level of HECW1 in ccRCC was evaluated by the H-score method. (**c**) A time-dependent receiver operating characteristic (ROC) analysis was used to determine the optimum cut-off value of HECW1 in the randomized training cohort (at a 3:2 ratio). (**d-g**) Kaplan-Meier curves for the overall survival (OS) and progression-free survival (PFS) of ccRCC patients were analyzed according to HECW1 expression in the randomized training cohort (d, e) and validation cohort (f, g) (at a 3:2 ratio)

To examine whether low HECW1 expression indicates the post-operative prognosis of ccRCC patients, ccRCC specimens from postoperative ccRCC patients (*n* = 300) were employed. These samples were randomly divided into a training and a validation cohort at a 3:2 ratio (Fig. 3a; Table 1). First, IHC assays were applied to examine the expression level of HECW1, and then a time-dependent receiver operating characteristic (ROC) analysis was used to show that the optimal cut-off value for dividing ccRCC patients from the training cohort into HECW1^low^ and HECW1^high^ groups was 105 (Fig. 3b-c; Table 2). As shown in Table 2, the HECW1^low^ group presented higher TNM stage, SSIGN score, and WHO/International Society of Urological Pathology (ISUP) grade. In addition, Kaplan-Meier survival analysis demonstrated that the HECW1^low^ group exhibited worse overall survival (OS) and progression-free survival (PFS) than the HECW1^high^ group (Fig. 3d-e). The validation cohort of ccRCC patients was employed to corroborate the above results using the cut-off value derived from the training cohort and showed that low expression of HECW1 indicated unfavorable clinicopathological features and short survival in ccRCC patients (Fig. 3f-g; Table 3). To confirm the above results, the ccRCC patients were also stochastically divided into a training cohort and a validation cohort at a 1:1 ratio (Fig. 3a; Table 1). The analysis of these cohorts demonstrated that the HECW1^low^ group showed advanced TNM stage, SSIGN score, and WHO/ISUP grade and shorter OS and PFS than the HECW1^high^ group both in the training cohort and in the validation cohort (Fig. S1a-f; Tables 4 and 5). These findings present that low HECW1 expression indicates unfavorable clinicopathological features and short survival in ccRCC patients.

**Table 2:** The correlation between HECW1 expression and clinicopathologic characteristics of patients with ccRCC in the training cohort (*n =* 180) (3:2 ratio)

Characteristics	HECW1		Sum(180)	***P*** value
	High expression(107)	Low expression(73)		
**Age**				1.000
< 60	65	45	110	
≥ 60	42	28	70	
**Gender**				0.095
Male	70	57	127	
Female	37	16	53	
**WHO/ISUP Grading**				**< 0.001**
I-II	93	44	137	
III-IV	14	29	43	
**TNM stage**				**< 0.001**
I-II	106	55	161	
III-IV	1	18	19	
**SSIGN**				**< 0.001**
1–4	106	62	168	
≥ 5	1	11	12	

*SSIGN* Stage, Size, Grade, and Necrosis, *TNM* Tumor Node Metastasis

**Table 3:** The correlation between HECW1 expression and clinicopathologic characteristics of patients with ccRCC in the validation cohort (*n =* 120) (3:2 ratio)

Characteristics	HECW1		Sum(120)	***P*** value
	High expression(77)	Low expression(43)		
**Age**				1
< 60	46	26	72	
≥ 60	31	17	48	
**Gender**				0.667
Male	55	33	88	
Female	22	10	32	
**WHO/ISUP Grading**				**0.002**
I-II	66	25	91	
III-IV	11	18	29	
**TNM stage**				**< 0.001**
I-II	74	31	105	
III-IV	3	12	15	
**SSIGN**				**0.002**
1–4	77	37	114	
≥ 5	0	6	6	

*SSIGN* Stage, Size, Grade, and Necrosis, *TNM* Tumor Node Metastasis

**Table 4:** The correlation between HECW1 expression and clinicopathologic characteristics of patients with ccRCC in the training cohort (*n =* 150) (1:1 ratio)

Characteristics	HECW1		Sum(150)	***P*** value
	High expression(90)	Low expression(60)		
**Age**				0.619
< 60	47	34	81	
≥ 60	43	26	69	
**Gender**				0.063
Male	61	49	110	
Female	29	11	40	
**WHO/ISUP Grading**				**< 0.001**
I-II	77	31	108	
III-IV	13	29	42	
**TNM stage**				**0.003**
I-II	87	49	136	
III-IV	3	11	14	
**SSIGN**				**< 0.001**
1–4	90	49	139	
≥ 5	0	11	11	

*SSIGN* Stage, Size, Grade, and Necrosis, *TNM* Tumor Node Metastasis

**Table 5:** The correlation between HECW1 expression and clinicopathologic characteristics of patients with ccRCC in the validation cohort (*n =* 150) (1:1 ratio)

Characteristics	HECW1		Sum(150)	***P*** value
	High expression(94)	Low expression(56)		
**Age**				0.858
< 60	64	37	101	
≥ 60	30	19	49	
**Gender**				0.582
Male	64	41	105	
Female	30	15	45	
**WHO/ISUP Grading**				**0.006**
I-II	82	38	120	
III-IV	12	18	30	
**TNM stage**				**< 0.001**
I-II	93	37	130	
III-IV	1	19	20	
**SSIGN**				**< 0.001**
1–4	93	49	142	
≥ 5	1	7	8	

*SSIGN* Stage, Size, Grade, and Necrosis, *TNM* Tumor Node Metastasis

### Combining intratumoral HECW1 expression and the clinical indicators exhibits higher prognostic accuracy in evaluating the postoperative prognosis of ccRCC patients

To further determine the prognostic value of HECW1 in ccRCC patients, univariate and multivariate Cox regression analyses were performed to examine whether HECW1 serves as an independent risk factor for predicting the OS and PFS of ccRCC patients. Even after multivariable adjustment of the clinical characteristics, HECW1 expression, TNM stage, and SSIGN score served as independent risk factors in both the training cohort (Tables 6 and 7) and the validation cohort (Tables 8 and 9) regardless of the ratio (3:2 or 1:1). Therefore, HECW1 expression in specimens serves as an independent risk factor for ccRCC patients’ prognosis.

**Table 6:** Univariate and multivariate cox regression analysis of HECW1 expression classifier and clinical characteristics with overall survival and progression-free survival in the training cohort (*n =* 180) (3:2ratio)

	Overall survival				Progression-free survival			
**Characteristics**	**Univariate**		**Multivariate**		**Univariate**		**Multivariate**	
	**HR(95% CI)**	***P*** **value**	**HR(95% CI)**	***P*** **value**	**HR(95% CI)**	***P*** **value**	**HR(95% CI)**	***P*** **value**
**Age (≥60y vs < 60y)**	1.061 (0.493–2.288)	0.879			0.664 (0.324–1.350)	0.664		
**Gender (Male vs Female)**	0.558 (0.211–1.476)	0.24			1.154 (0.577–2.31)	0.686		
**WHO/ISUP Grading (III-IV vs I-II)**	3.901 (1.813–8.394)	**< 0.001**	2.095 (0.889–4.938)	0.091	2.345 (1.205–4.561)	**0.012**	1.065 (0.521–2.177)	0.863
**TNM stage (III-IV vs I-II)**	11.791 (5.126–27.121)	**< 0.001**	3.797 (1.633–8.829)	**0.002**	9.999 (5.004–19.982)	**< 0.001**	4.504 (2.145–9.457)	**< 0.001**
**SSIGN (≥5 vs 1–4)**	10.030 (4.199–23.961)	**< 0.001**	3.872 (1.477–10.150)	**0.006**	19.698 (9.141–42.449)	**< 0.001**	7.815 (3.456–17.672)	**< 0.001**
**HECW1 (High vs Low)**	69 (9.253–514.551)	**< 0.001**	33.384 (4.218–264.224)	**< 0.001**	15.432 (5.919–40.233)	**< 0.001**	7.554 (2.602–21.930)	**< 0.001**

*SSIGN* Stage, Size, Grade, and Necrosis, *TNM* Tumor Node Metastasis

**Table 7:** Univariate and multivariate cox regression analysis of HECW1 expression classifier and clinical characteristics with overall survival and progression-free survival in the validation cohort (*n =* 120) (3:2ratio)

	Overall survival				Progression-free survival			
**Characteristics**	**Univariate**		**Multivariate**		**Univariate**		**Multivariate**	
	**HR(95% CI)**	***P*** **value**	**HR(95% CI)**	***P*** **value**	**HR(95% CI)**	***P*** **value**	**HR(95% CI)**	***P*** **value**
**Age (≥60y vs < 60y)**	1.535 (0.690–3.417)	0.294			2.417 (1.052–5.554)	**0.038**		
**Gender (Male vs Female)**	1.274 (0.542–2.994)	0.579			0.807 (0.316–2.061)	0.654		
**WHO/ISUP Grading (III-IV vs I-II)**	3.064 (1.396–6.723)	**0.005**	1.029 (0.418–2.536)	0.95	3.484 (1.534–7.912)	**0.003**	1.180 (0.477–2.923)	0.72
**TNM stage (III-IV vs I-II)**	5.160 (2.272–11.719)	**< 0.001**	3.248 (1.171–9.011)	**0.024**	5.287 (2.292–12.195)	**< 0.001**	3.087 (1.052–9.060)	**0.04**
**SSIGN (≥5 vs 1–4)**	5.492 (2.135–14.130)	**< 0.001**	3.425 (1.025–11.443)	**0.045**	10.988 (3.921–30.795)	**< 0.001**	5.918 (1.383–9.060)	**0.017**
**HECW1 (High vs Low)**	13.743 (4.100–46.065)	**< 0.001**	7.480 (1.978–28.295)	**0.003**	21.144 (4.947–90.383)	**< 0.001**	10.613 (2.226–50.600)	**0.003**

*SSIGN* Stage, Size, Grade, and Necrosis, *TNM* Tumor Node Metastasis

**Table 8:** Univariate and multivariate cox regression analysis of HECW1 expression classifier and clinical characteristics with overall survival and progression-free survival in the training cohort (*n =* 150) (1:1 ratio)

	Overall survival				Progression-free survival			
**Characteristics**	**Univariate**		**Multivariate**		**Univariate**		**Multivariate**	
	**HR(95% CI)**	***P*** **value**	**HR(95% CI)**	***P*** **value**	**HR(95% CI)**	***P*** **value**	**HR(95% CI)**	***P*** **value**
**Age (≥60y vs < 60y)**	1.550 (0.681–3.525)	0.296			1.251 (0.599–2.615)	0.551		
**Gender (Male vs Female)**	0.995 (0.409–2.424)	0.992			0.958 (0.435–2.110)	0.915		
**WHO/ISUP Grading (III-IV vs I-II)**	3.804 (1.645–8.795)	**0.002**	1.412 (0.595–3.352)	0.434	2.148 (1.032–4.469)	**0.041**	1.079 (0.499–2.333)	0.846
**TNM stage (III-IV vs I-II)**	5.843 (2.222–15.363)	**< 0.001**	3.028 (1.115–8.222)	**0.03**	9.042 (4.157–19.667)	**< 0.001**	5.441 (2.329–12.712)	**< 0.001**
**SSIGN (≥5 vs 1–4)**	9.235 (3.881–21.975)	**< 0.001**	2.635 (1.058–6.562)	**0.037**	17.907 (8.057–39.800)	**< 0.001**	7.604 (3.169–18.243)	**< 0.001**
**HECW1 (High vs Low)**	51.099 (6.841–381.667)	**< 0.001**	29.907 (3.750–238.530)	**0.001**	10.054 (4.183–26.373)	**< 0.001**	5.072 (1.728–14.891)	**0.003**

*SSIGN* Stage, Size, Grade, and Necrosis, *TNM* Tumor Node Metastasis

**Table 9:** Univariate and multivariate cox regression analysis of HECW1 expression classifier and clinical characteristics with overall survival and progression-free survival in the validation cohort (*n =* 150) (1:1 ratio)

	Overall survival				Progression-free survival			
**Characteristics**	**Univariate**		**Multivariate**		**Univariate**		**Multivariate**	
	**HR(95% CI)**	***P*** **value**	**HR(95% CI)**	***P*** **value**	**HR(95% CI)**	***P*** **value**	**HR(95% CI)**	***P*** **value**
**Age (≥60y vs < 60y)**	1.037 (0.480–2.244)	0.926			0.978 (0.455–2.101)	0.955		
**Gender (Male vs Female)**	0.729 (0.296–1.796)	0.492			1.117 (0.510–2.450)	0.782		
**WHO/ISUP Grading (III-IV vs I-II)**	3.954 (1.873–8.345)	**< 0.001**	1.568 (0.657–3.745)	0.311	3.783 (1.832–7.811)	**< 0.001**	1.530 (0.673–3.478)	0.31
**TNM stage (III-IV vs I-II)**	9.069 (4.307–19.096)	**< 0.001**	3.832 (1.437–10.223)	**0.007**	7.319 (3.550–15.093)	**< 0.001**	2.503 (1.055–5.940)	**0.037**
**SSIGN (≥5 vs 1–4)**	7.223 (2.727–19.136)	**< 0.001**	5.953 (1.773–19.982)	**0.004**	15.156 (5.370–42.772)	**< 0.001**	9.803 (2.993–32.113)	**< 0.001**
**HECW1 (High vs Low)**	19.480 (5.883–64.500)	**< 0.001**	7.483 (1.897–29.514)	**0.004**	64.549 (8.782–474.440)	**< 0.001**	31.602 (3.989–250.386)	**0.001**

*SSIGN* Stage, Size, Grade, and Necrosis, *TNM* Tumor Node Metastasis

Furthermore, we examined the prognostic accuracy of combining HECW1 expression with a current prognostic indicator, TNM stage or SSIGN score, in predicting ccRCC patient prognosis. Then, time-dependent concordance index (c-index) analysis was used in the training cohort (at a 3:2 ratio), which demonstrated that combining HECW1 and TNM stage or SSIGN score exhibited a higher c-index value than any of these indicators alone in predicting ccRCC patients’ prognosis (Table 10). The above results were also confirmed in the validation cohort (at a 3:2 ratio) and the training cohort and validation cohort (at a 1:1 ratio) (Tables 10 and 11). Taken together, these findings indicate that improved prognostic accuracy in predicting the postoperative prognosis of ccRCC patients can be accomplished by combining intratumoral HECW1 expression and existing clinical indicators.

**Table 10:** C-index analysis of the prognostic accuracy of HECW1 and other variables for overall survival and progression-free survival in the training cohort (*n =* 180) and validation cohort (*n =* 120) (3:2 ratio)

Characteristics	Overall survival		Progression-free survival	
	training cohort(***n =*** 180)	validation cohort(***n =*** 120)	training cohort(***n =*** 180)	validation cohort(***n =*** 120)
**TNM stage**	0.700(0.601–0.799)	0.608(0.512–0.704)	0.699(0.616–0.782)	0.606(0.469–0.607)
**SSIGN**	0.647(0.549–0.745)	0.608(0.511–0.705)	0.684(0.598–0.770)	0.624(0.536–0.728)
**HECW1**	0.825(0.790–0.860)	0.807(0.755–0.859)	0.791(0.749–0.833)	0.828(0.786–0.874)
**HECW1 + TNM**	0.876(0.833–0.919)	0.822(0.768–0.876)	0.842(0.796–0.888)	0.836(0.761–0.863)
**HECW1 + SSIGN**	0.870(0.825–0.915)	0.830(0.771–0.889)	0.854(0.803–0.905)	0.857(0.817–0.911)

*SSIGN* Stage, Size, Grade, and Necrosis, *TNM* Tumor Node Metastasis

**Table 11:** C-index analysis of the prognostic accuracy of HECW1 and other variables for overall survival and progression-free survival in the training cohort (*n =* 150) and validation cohort (*n =* 150) (1:1 ratio)

Characteristics	Overall survival		Progression-free survival	
	training cohort(***n =*** 150)	validation cohort(***n =*** 150)	training cohort(***n =*** 150)	validation cohort(***n =*** 150)
**TNM stage**	0.669(0.588–0.750)	0.686(0.588–0.784)	0.659(0.566–0.752)	0.664(0.572–0.756)
**SSIGN**	0.684(0.598–0.770)	0.605(0.513–0.697)	0.734(0.637–0.831)	0.607(0.521–0.692)
**HECW1**	0.791(0.749–0.833)	0.817(0.776–0.858)	0.781(0.731–0.831)	0.830(0.789–0.871)
**HECW1 + TNM**	0.834(0.788–0.880)	0.847(0.799–0.895)	0.829(0.775–0.883)	0.845(0.797–0.893)
**HECW1 + SSIGN**	0.854(0.803–0.905)	0.844(0.794–0.894)	0.854(0.793–0.915)	0.861(0.816–0.906)

*SSIGN* Stage, Size, Grade, and Necrosis, *TNM* Tumor Node Metastasis

## Discussion

The identification of reliable and helpful indicators for evaluating ccRCC patient disease progression and prognosis is crucial for improving clinical therapies and patient survival [16]. Although numerous studies have reported that tumor biomarkers predict ccRCC patient prognosis, combining intratumoral markers and clinical indicators could predict ccRCC patient prognosis more accurately than any of these markers alone [17, 18]. Our present study integrates HECW1 expression into a prognostic model with the TNM stage or SSIGN score, which results in better accuracy in evaluating ccRCC patient prognosis than that achieved with only one of these indicators.

Given its role in DNA damage responses and p53-mediated apoptotic cell death, it is possible that the abnormal expression of HECW1 may disrupt cell homeostasis and cause tumorigenesis [19, 20]. Recently, mutations of HECW1 have been identified in non-small cell lung cancer and muscle-invasive transitional cell carcinoma [10, 11]. Additionally, HECW1 has been found to negatively regulate ErbB4 protein expression via ubiquitin-mediated degradation in breast cancer [9]. In our present study, the expression of HECW1 in ccRCC was analyzed in TCGA and compared between matched postoperative ccRCC specimens and normal adjacent tissues in our clinical center. Altogether, these findings showed that the expression of HECW1 is commonly downregulated in ccRCC. Although the results of immunohistochemical studies showed that HECW1 expression was higher in 10 muscle-invasive transitional cell carcinomas than in normal adjacent samples [11], its expression may be based on the tumor type. The results of the present study also revealed that a low expression of HECW1 was associated with a high TNM stage, bone metastasis, and targeted drug resistance in ccRCC. In addition, the biological function of HECW1 was reported only recently in a paper showing that HECW1 promoted the proliferation, migration and invasion of non-small cell lung cancer cells. However, to elucidate the function of HECW1 in other malignant tumors, including ccRCC, additional studies are needed in the future.

The prognostic value of HECW1 has not been reported in other types of tumors. For the first time, we demonstrated that low HECW1 expression indicated higher TNM stage, SSIGN score, WHO/ISUP grade, and poor prognosis in ccRCC patients. Although many prognostic biomarkers such as oncogenes, tumor suppressive genes, and tumor-infiltrating immune cells have been reported in evaluations of the postoperative prognosis of ccRCC patients [16], they have not been applicable to clinical practice. One of the reasons may be that these indicators have not been compared and integrated into established clinical prognostic models, such as the TNM staging system. Our present study not only demonstrated that HECW1 is a potential biomarker for ccRCC patient disease progression and survival rate but also compared the accuracy of combining HECW1 expression with current prognostic indicators, namely, the TNM stage or SSIGN score, with the accuracy of these indicators alone for predicting ccRCC patient prognosis. We found that combining HECW1 and TNM stage or SSIGN score presented a higher c-index value than any of these indicators alone for predicting ccRCC patient prognosis. Therefore, improved prognostic accuracy can be achieved by combining intratumoral HECW1 expression and clinical indicators when evaluating ccRCC patients’ postoperative prognosis. However, ccRCC patients from only one clinical center were employed in this study. Our future studies will include more ccRCC patients from other clinical centers.

## Supplementary Information

**Additional file 1.**

## Data Availability

Datasets from The Cancer Genome Atlas (TCGA) were downloaded from National Cancer Institute GDC Data Portal (https://portal.gdc.cancer.gov) using FirebrowseR package. The data used and analyzed in this study are available from the corresponding authors on reasonable request.
