# Supplementary material for: Integrating HECW1 expression into the clinical indicators exhibits high accuracy in assessing the prognosis of patients with clear cell renal cell carcinoma
Source: BMC Cancer. 2021 Aug 4;21:890. doi: 10.1186/s12885-021-08631-9 (PMC8335872; doi:10.1186/s12885-021-08631-9)
Supplement: Supplementary file 1 — Additional file 1. [file 12885_2021_8631_MOESM1_ESM.doc]

**Supplementary Materials**


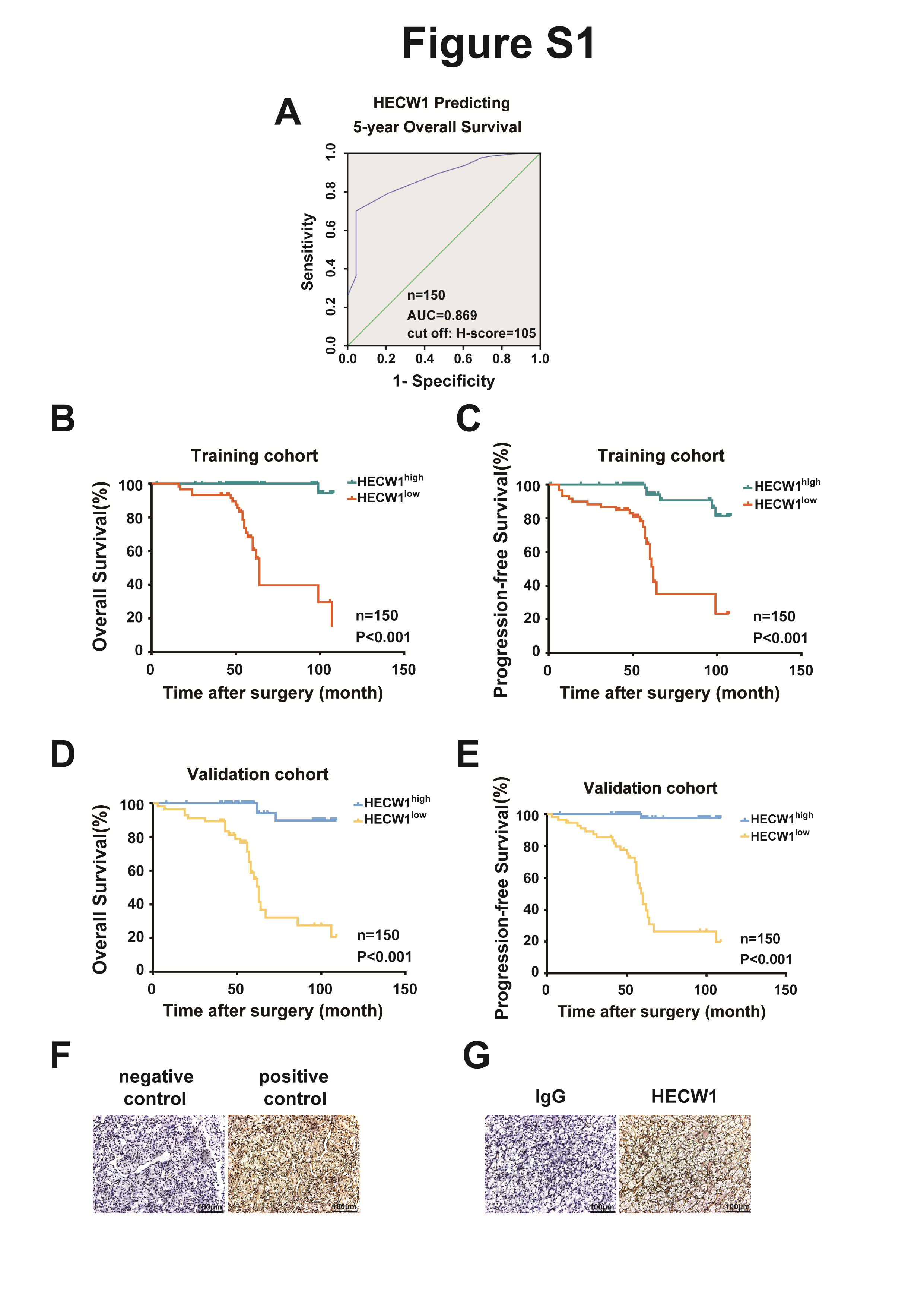


**Figure S1 Low HECW1 expression is predictive of unfavorable clinicopathological characteristics and poor postoperative prognosis in ccRCC patients.** (a) A time-dependent ROC analysis was used to determine the optimum cut-off value of HECW1 in the training cohort (at a 1:1 ratio). (b-e) Kaplan-Meier curves for the OS and PFS of ccRCC patients were analyzed according to HECW1 expression in the randomized training cohort (b, c) and validation cohort (d, e) (at a 1:1 ratio). (f-g) Representative IHC image of isotype, positive, and negative controls of HECW1 (scale bar = 100 μm).

**Supplementary Table** S1

| **Patients** | **The H score of patients with clear cell renal cell carcinoma (ccRCC)** | | | | | | | | | | | | | |
| --- | --- | --- | --- | --- | --- | --- | --- | --- | --- | --- | --- | --- | --- | --- |
|  | **Normal** | | | | | | | **Tumor** | | | | | | |
|  | 1 | | 2 | | 3 | | Mean | 1 | | 2 | | 3 | | Mean |
| 1 | 0*30+1*0+2*70+3*0 | 140 | 0*40+1*10+2*50+3*0 | 110 | 0*40+1*10+2*50+3*0 | 110 | 120 | 0*40+1*60+2*0+3*0 | 60 | 0*20+1*80+2*0+3*0 | 80 | 0*30+1*70+2*0+3*0 | 70 | 70 |
| 2 | 0*40+1*0+2*60+3*0 | 120 | 0*40+1*0+2*60+3*0 | 120 | 0*40+1*0+2*60+3*0 | 120 | 120 | 0*30+1*70+2*0+3*0 | 70 | 0*20+1*80+2*0+3*0 | 80 | 0*30+1*20+2*50+3*0 | 120 | 90 |
| 3 | 0*20+1*10+2*70+3*0 | 150 | 0*10+1*10+2*80+3*0 | 170 | 0*0+1*10+2*90+3*0 | 190 | 170 | 0*30+1*70+2*0+3*0 | 70 | 0*30+1*70+2*0+3*0 | 70 | 0*30+1*70+2*0+3*0 | 70 | 70 |
| 4 | 0*10+1*10+2*0+3*80 | 250 | 0*20+1*0+2*0+3*80 | 240 | 0*0+1*0+2*100+3*0 | 200 | 230 | 0*20+1*10+2*70+3*0 | 150 | 0*10+1*10+2*80+3*0 | 170 | 0*0+1*100+2*0+3*0 | 100 | 140 |
| 5 | 0*20+1*10+2*70+3*0 | 150 | 0*20+1*10+2*70+3*0 | 150 | 0*20+1*10+2*70+3*0 | 150 | 150 | 0*0+1*100+2*0+3*0 | 100 | 0*40+1*10+2*50+3*0 | 110 | 0*40+1*0+2*60+3*0 | 120 | 110 |
| 6 | 0*20+1*0+2*80+3*0 | 160 | 0*30+1*0+2*70+3*0 | 140 | 0*40+1*0+2*60+3*0 | 120 | 140 | 0*40+1*0+2*60+3*0 | 120 | 0*50+1*0+2*50+3*0 | 100 | 0*10+1*10+2*80+3*0 | 170 | 130 |
| 7 | 0*10+1*90+2*0+3*0 | 90 | 0*0+1*100+2*0+3*0 | 100 | 0*40+1*10+2*50+3*0 | 110 | 100 | 0*40+1*10+2*50+3*0 | 110 | 0*40+1*10+2*50+3*0 | 110 | 0*40+1*10+2*50+3*0 | 110 | 110 |
| 8 | 0*0+1*20+2*0+3*80 | 260 | 0*20+1*0+2*0+3*80 | 240 | 0*20+1*10+2*0+3*70 | 220 | 240 | 0*20+1*80+2*0+3*0 | 80 | 0*20+1*80+2*0+3*0 | 80 | 0*40+1*10+2*50+3*0 | 110 | 90 |
| 9 | 0*40+1*0+2*60+3*0 | 120 | 0*40+1*0+2*60+3*0 | 120 | 0*40+1*0+2*60+3*0 | 120 | 120 | 0*10+1*90+2*0+3*0 | 90 | 0*10+1*90+2*0+3*0 | 90 | 0*10+1*90+2*0+3*0 | 90 | 90 |
| 10 | 0*0+1*0+2*100+3*0 | 200 | 0*0+1*10+2*90+3*0 | 190 | 0*30+1*0+2*0+3*70 | 210 | 200 | 0*20+1*80+2*0+3*0 | 80 | 0*10+1*90+2*0+3*0 | 90 | 0*0+1*100+2*0+3*0 | 100 | 90 |
| 11 | 0*10+1*90+2*0+3*0 | 90 | 0*0+1*100+2*0+3*0 | 100 | 0*40+1*10+2*50+3*0 | 110 | 100 | 0*50+1*50+2*0+3*0 | 50 | 0*40+1*60+2*0+3*0 | 60 | 0*30+1*70+2*0+3*0 | 70 | 60 |
| 12 | 0*20+1*10+2*70+3*0 | 150 | 0*20+1*0+2*80+3*0 | 160 | 0*30+1*0+2*70+3*0 | 140 | 150 | 0*20+1*80+2*0+3*0 | 80 | 0*10+1*90+2*0+3*0 | 90 | 0*0+1*100+2*0+3*0 | 100 | 90 |
| 13 | 0*10+1*0+2*0+3*90 | 270 | 0*10+1*0+2*0+3*90 | 270 | 0*10+1*0+2*0+3*90 | 270 | 270 | 0*20+1*0+2*0+3*80 | 240 | 0*30+1*0+2*0+3*70 | 210 | 0*30+1*0+2*0+3*70 | 210 | 220 |
| 14 | 0*10+1*0+2*0+3*90 | 270 | 0*0+1*20+2*0+3*80 | 260 | 0*10+1*10+2*0+3*80 | 250 | 260 | 0*50+1*50+2*0+3*0 | 50 | 0*50+1*50+2*0+3*0 | 50 | 0*50+1*50+2*0+3*0 | 50 | 50 |
| 15 | 0*20+1*0+2*0+3*80 | 240 | 0*0+1*20+2*0+3*80 | 260 | 0*10+1*10+2*0+3*80 | 250 | 250 | 0*50+1*50+2*0+3*0 | 50 | 0*30+1*70+2*0+3*0 | 70 | 0*10+1*90+2*0+3*0 | 90 | 70 |
| 16 | 0*0+1*0+2*10+3*90 | 290 | 0*0+1*0+2*20+3*80 | 280 | 0*10+1*0+2*0+3*90 | 270 | 280 | 0*0+1*0+2*20+3*80 | 120 | 0*20+1*0+2*80+3*0 | 160 | 0*30+1*0+2*70+3*0 | 140 | 140 |
| 17 | 0*20+1*10+2*0+3*70 | 220 | 0*20+1*10+2*0+3*70 | 220 | 0*20+1*10+2*0+3*70 | 220 | 220 | 0*40+1*10+2*50+3*0 | 110 | 0*40+1*10+2*50+3*0 | 110 | 0*40+1*10+2*50+3*0 | 110 | 110 |
| 18 | 0*30+1*0+2*0+3*70 | 210 | 0*20+1*0+2*0+3*80 | 240 | 0*10+1*0+2*0+3*90 | 270 | 240 | 0*30+1*70+2*0+3*0 | 70 | 0*20+1*80+2*0+3*0 | 80 | 0*20+1*10+2*70+3*0 | 150 | 100 |
| 19 | 0*10+1*10+2*0+3*80 | 250 | 0*20+1*0+2*10+3*70 | 230 | 0*30+1*0+2*0+3*70 | 210 | 230 | 0*70+1*30+2*0+3*0 | 30 | 0*70+1*30+2*0+3*0 | 30 | 0*40+1*60+2*0+3*0 | 60 | 40 |
| 20 | 0*10+1*0+2*0+3*90 | 270 | 0*10+1*10+2*0+3*80 | 250 | 0*0+1*0+2*10+3*90 | 290 | 270 | 0*10+1*0+2*90+3*0 | 180 | 0*10+1*10+2*80+3*0 | 170 | 0*20+1*0+2*80+3*0 | 160 | 170 |
| 21 | 0*30+1*0+2*70+3*0 | 140 | 0*30+1*0+2*70+3*0 | 140 | 0*30+1*0+2*70+3*0 | 140 | 140 | 0*20+1*10+2*70+3*0 | 150 | 0*20+1*0+2*80+3*0 | 160 | 0*20+1*0+2*80+3*0 | 170 | 160 |
| 22 | 0*0+1*0+2*100+3*0 | 200 | 0*10+1*10+2*80+3*0 | 170 | 0*10+1*10+2*80+3*0 | 170 | 180 | 0*20+1*10+2*70+3*0 | 150 | 0*20+1*10+2*70+3*0 | 150 | 0*20+1*10+2*70+3*0 | 150 | 150 |
| 23 | 0*20+1*0+2*10+3*70 | 230 | 0*20+1*0+2*10+3*70 | 230 | 0*20+1*0+2*10+3*70 | 230 | 230 | 0*40+1*0+2*60+3*0 | 120 | 0*40+1*10+2*50+3*0 | 110 | 0*20+1*0+2*80+3*0 | 160 | 130 |
| 24 | 0*0+1*0+2*100+3*0 | 200 | 0*40+1*0+2*60+3*0 | 170 | 0*0+1*0+2*100+3*0 | 200 | 190 | 0*10+1*0+2*90+3*0 | 180 | 0*40+1*10+2*50+3*0 | 110 | 0*20+1*0+2*80+3*0 | 160 | 150 |
| 25 | 0*0+1*0+2*100+3*0 | 200 | 0*40+1*0+2*0+3*60 | 180 | 0*10+1*10+2*0+3*80 | 250 | 210 | 0*40+1*10+2*50+3*0 | 110 | 0*30+1*10+2*60+3*0 | 130 | 0*10+1*90+2*0+3*0 | 90 | 110 |
| 26 | 0*10+1*10+2*0+3*80 | 250 | 0*0+1*0+2*20+3*80 | 280 | 0*20+1*10+2*0+3*70 | 220 | 250 | 0*40+1*10+2*50+3*0 | 110 | 0*20+1*0+2*80+3*0 | 160 | 0*10+1*0+2*90+3*0 | 180 | 150 |
| 27 | 0*10+1*10+2*80+3*0 | 170 | 0*10+1*10+2*80+3*0 | 170 | 0*10+1*10+2*80+3*0 | 170 | 170 | 0*10+1*10+2*80+3*0 | 170 | 0*10+1*10+2*80+3*0 | 170 | 0*10+1*10+2*80+3*0 | 170 | 170 |
| 28 | 0*0+1*0+2*100+3*0 | 200 | 0*0+1*0+2*100+3*0 | 200 | 0*0+1*0+2*100+3*0 | 200 | 200 | 0*0+1*100+2*0+3*0 | 100 | 0*40+1*0+2*60+3*0 | 120 | 0*40+1*10+2*50+3*0 | 110 | 110 |
| 29 | 0*0+1*0+2*100+3*0 | 200 | 0*10+1*0+2*0+3*90 | 270 | 0*0+1*0+2*20+3*80 | 280 | 250 | 0*10+1*10+2*80+3*0 | 170 | 0*10+1*10+2*80+3*0 | 170 | 0*10+1*10+2*80+3*0 | 170 | 170 |
| 30 | 0*70+1*30+2*0+3*0 | 30 | 0*70+1*30+2*0+3*0 | 30 | 0*70+1*30+2*0+3*0 | 30 | 30 | 0*20+1*10+2*70+3*0 | 150 | 0*20+1*10+2*70+3*0 | 150 | 0*20+1*10+2*70+3*0 | 150 | 150 |
| 31 | 0*30+1*0+2*0+3*70 | 210 | 0*20+1*0+2*0+3*80 | 240 | 0*40+1*0+2*0+3*60 | 180 | 210 | 0*40+1*10+2*50+3*0 | 110 | 0*30+1*0+2*70+3*0 | 140 | 0*30+1*0+2*70+3*0 | 140 | 130 |
| 32 | 0*50+1*0+2*50+3*0 | 100 | 0*50+1*0+2*50+3*0 | 100 | 0*50+1*0+2*50+3*0 | 100 | 100 | 0*10+1*10+2*80+3*0 | 170 | 0*10+1*10+2*80+3*0 | 170 | 0*10+1*10+2*80+3*0 | 170 | 170 |
| 33 | 0*50+1*50+2*0+3*0 | 50 | 0*10+1*90+2*0+3*0 | 90 | 0*50+1*0+2*50+3*0 | 100 | 80 | 0*20+1*80+2*0+3*0 | 80 | 0*10+1*90+2*0+3*0 | 90 | 0*0+1*100+2*0+3*0 | 100 | 90 |
| 34 | 0*0+1*0+2*100+3*0 | 200 | 0*0+1*0+2*100+3*0 | 200 | 0*10+1*10+2*80+3*0 | 170 | 190 | 0*40+1*10+2*50+3*0 | 110 | 0*40+1*10+2*50+3*0 | 110 | 0*40+1*10+2*50+3*0 | 110 | 110 |
| 35 | 0*20+1*0+2*10+3*70 | 230 | 0*20+1*0+2*10+3*70 | 230 | 0*20+1*0+2*10+3*70 | 230 | 230 | 0*20+1*0+2*80+3*0 | 160 | 0*10+1*0+2*90+3*0 | 180 | 0*0+1*0+2*100+3*0 | 200 | 180 |
| 36 | 0*50+1*0+2*50+3*0 | 100 | 0*50+1*0+2*50+3*0 | 100 | 0*50+1*0+2*50+3*0 | 100 | 100 | 0*0+1*0+2*100+3*0 | 200 | 0*0+1*20+2*0+3*80 | 260 | 0*0+1*0+2*100+3*0 | 200 | 220 |
| 37 | 0*10+1*0+2*0+3*90 | 270 | 0*20+1*0+2*10+3*70 | 230 | 0*10+1*10+2*0+3*80 | 250 | 250 | 0*50+1*0+2*0+3*50 | 150 | 0*50+1*0+2*0+3*50 | 150 | 0*50+1*0+2*0+3*50 | 150 | 150 |
| 38 | 0*30+1*0+2*70+3*0 | 140 | 0*30+1*0+2*70+3*0 | 140 | 0*30+1*0+2*70+3*0 | 140 | 140 | 0*20+1*0+2*10+3*70 | 230 | 0*20+1*0+2*10+3*70 | 230 | 0*20+1*0+2*10+3*70 | 230 | 230 |
| 39 | 0*40+1*0+2*0+3*60 | 180 | 0*40+1*0+2*0+3*60 | 180 | 0*40+1*0+2*0+3*60 | 180 | 180 | 0*0+1*100+2*0+3*0 | 100 | 0*0+1*100+2*0+3*0 | 100 | 0*0+1*100+2*0+3*0 | 100 | 100 |
| 40 | 0*0+1*0+2*100+3*0 | 200 | 0*0+1*10+2*90+3*0 | 190 | 0*20+1*0+2*0+3*80 | 240 | 210 | 0*50+1*50+2*0+3*0 | 50 | 0*50+1*50+2*0+3*0 | 50 | 0*50+1*50+2*0+3*0 | 50 | 50 |
| 41 | 0*10+1*10+2*80+3*0 | 170 | 0*10+1*10+2*80+3*0 | 170 | 0*10+1*10+2*80+3*0 | 170 | 170 | 0*0+1*0+2*100+3*0 | 200 | 0*30+1*0+2*0+3*70 | 210 | 0*10+1*10+2*0+3*80 | 250 | 220 |
| 42 | 0*20+1*0+2*0+3*80 | 240 | 0*20+1*0+2*0+3*80 | 240 | 0*20+1*0+2*0+3*80 | 240 | 240 | 0*10+1*0+2*90+3*0 | 180 | 0*10+1*0+2*90+3*0 | 180 | 0*10+1*0+2*90+3*0 | 180 | 180 |
| 43 | 0*30+1*0+2*70+3*0 | 140 | 0*30+1*0+2*70+3*0 | 140 | 0*30+1*0+2*70+3*0 | 140 | 140 | 0*10+1*0+2*90+3*0 | 180 | 0*10+1*0+2*90+3*0 | 180 | 0*10+1*0+2*90+3*0 | 180 | 180 |
| 44 | 0*10+1*0+2*0+3*90 | 270 | 0*10+1*0+2*0+3*90 | 270 | 0*10+1*0+2*0+3*90 | 270 | 270 | 0*50+1*50+2*0+3*0 | 50 | 0*50+1*50+2*0+3*0 | 50 | 0*50+1*50+2*0+3*0 | 50 | 50 |
| 45 | 0*20+1*0+2*0+3*80 | 240 | 0*20+1*0+2*0+3*80 | 240 | 0*20+1*0+2*0+3*80 | 240 | 240 | 0*10+1*0+2*90+3*0 | 180 | 0*10+1*0+2*90+3*0 | 180 | 0*10+1*0+2*90+3*0 | 180 | 180 |
| 46 | 0*20+1*0+2*0+3*80 | 240 | 0*30+1*0+2*0+3*70 | 210 | 0*20+1*0+2*0+3*80 | 240 | 230 | 0*20+1*80+2*0+3*0 | 80 | 0*20+1*80+2*0+3*0 | 80 | 0*20+1*80+2*0+3*0 | 80 | 80 |
| 47 | 0*20+1*0+2*0+3*80 | 240 | 0*20+1*0+2*0+3*80 | 240 | 0*20+1*0+2*0+3*80 | 240 | 240 | 0*10+1*0+2*90+3*0 | 180 | 0*10+1*0+2*90+3*0 | 180 | 0*10+1*0+2*90+3*0 | 180 | 180 |
| 48 | 0*30+1*0+2*70+3*0 | 140 | 0*30+1*0+2*70+3*0 | 140 | 0*30+1*0+2*70+3*0 | 140 | 140 | 0*0+1*100+2*0+3*0 | 100 | 0*30+1*10+2*60+3*0 | 130 | 0*0+1*100+2*0+3*0 | 100 | 110 |
| 49 | 0*30+1*0+2*0+3*70 | 210 | 0*30+1*0+2*0+3*70 | 210 | 0*30+1*0+2*0+3*70 | 210 | 210 | 0*10+1*0+2*90+3*0 | 180 | 0*10+1*0+2*90+3*0 | 180 | 0*10+1*0+2*90+3*0 | 180 | 180 |
| 50 | 0*40+1*0+2*60+3*0 | 120 | 0*20+1*10+2*70+3*0 | 150 | 0*40+1*0+2*60+3*0 | 120 | 130 | 0*30+1*70+2*0+3*0 | 70 | 0*30+1*70+2*0+3*0 | 70 | 0*30+1*70+2*0+3*0 | 70 | 70 |
| 51 | 0*10+1*0+2*0+3*90 | 270 | 0*10+1*0+2*0+3*90 | 270 | 0*10+1*0+2*0+3*90 | 270 | 270 | 0*30+1*0+2*0+3*70 | 210 | 0*10+1*0+2*0+3*90 | 270 | 0*10+1*0+2*0+3*90 | 270 | 250 |
| 52 | 0*0+1*0+2*0+3*100 | 300 | 0*0+1*0+2*0+3*100 | 300 | 0*0+1*0+2*0+3*100 | 300 | 300 | 0*30+1*0+2*70+3*0 | 140 | 0*20+1*10+2*70+3*0 | 150 | 0*20+1*10+2*0+3*70 | 220 | 170 |
| 53 | 0*20+1*0+2*0+3*80 | 240 | 0*0+1*20+2*0+3*80 | 260 | 0*0+1*10+2*0+3*90 | 280 | 260 | 0*0+1*100+2*0+3*0 | 100 | 0*0+1*100+2*0+3*0 | 100 | 0*0+1*100+2*0+3*0 | 100 | 100 |
| 54 | 0*0+1*0+2*100+3*0 | 200 | 0*0+1*0+2*100+3*0 | 200 | 0*10+1*10+2*80+3*0 | 170 | 190 | 0*0+1*100+2*0+3*0 | 100 | 0*0+1*100+2*0+3*0 | 100 | 0*30+1*10+2*60+3*0 | 130 | 110 |
| 55 | 0*10+1*10+2*0+3*80 | 250 | 0*10+1*10+2*0+3*80 | 250 | 0*10+1*10+2*0+3*80 | 250 | 250 | 0*30+1*0+2*0+3*70 | 210 | 0*30+1*0+2*0+3*70 | 210 | 0*30+1*0+2*0+3*70 | 210 | 210 |
| 56 | 0*0+1*0+2*0+3*100 | 300 | 0*0+1*0+2*0+3*100 | 300 | 0*0+1*0+2*0+3*100 | 300 | 300 | 0*30+1*0+2*0+3*70 | 210 | 0*30+1*0+2*0+3*70 | 210 | 0*30+1*0+2*0+3*70 | 210 | 210 |
| 57 | 0*10+1*0+2*0+3*90 | 270 | 0*0+1*0+2*0+3*100 | 300 | 0*0+1*0+2*0+3*100 | 300 | 290 | 0*0+1*100+2*0+3*0 | 100 | 0*0+1*100+2*0+3*0 | 100 | 0*0+1*100+2*0+3*0 | 100 | 100 |
| 58 | 0*30+1*0+2*0+3*70 | 210 | 0*30+1*0+2*0+3*70 | 210 | 0*30+1*0+2*0+3*70 | 210 | 210 | 0*0+1*100+2*0+3*0 | 100 | 0*0+1*100+2*0+3*0 | 100 | 0*0+1*100+2*0+3*0 | 100 | 100 |
| 59 | 0*40+1*0+2*0+3*60 | 180 | 0*40+1*0+2*0+3*60 | 180 | 0*40+1*0+2*0+3*60 | 180 | 180 | 0*20+1*10+2*70+3*0 | 150 | 0*20+1*10+2*70+3*0 | 150 | 0*30+1*0+2*0+3*70 | 210 | 170 |
| 60 | 0*20+1*10+2*70+3*0 | 150 | 0*20+1*10+2*70+3*0 | 150 | 0*20+1*10+2*70+3*0 | 150 | 150 | 0*10+1*0+2*90+3*0 | 180 | 0*0+1*0+2*100+3*0 | 200 | 0*30+1*10+2*60+3*0 | 130 | 170 |
| 61 | 0*20+1*0+2*80+3*0 | 160 | 0*20+1*0+2*80+3*0 | 160 | 0*20+1*0+2*80+3*0 | 160 | 160 | 0*0+1*0+2*100+3*0 | 200 | 0*10+1*0+2*90+3*0 | 180 | 0*0+1*10+2*90+3*0 | 190 | 190 |
| 62 | 0*10+1*10+2*80+3*0 | 170 | 0*10+1*10+2*80+3*0 | 170 | 0*10+1*10+2*80+3*0 | 170 | 170 | 0*10+1*10+2*80+3*0 | 170 | 0*10+1*10+2*80+3*0 | 170 | 0*10+1*10+2*80+3*0 | 170 | 170 |
| 63 | 0*0+1*0+2*20+3*80 | 280 | 0*10+1*10+2*0+3*80 | 250 | 0*10+1*10+2*0+3*80 | 250 | 260 | 0*10+1*90+2*0+3*0 | 90 | 0*10+1*90+2*0+3*0 | 90 | 0*10+1*90+2*0+3*0 | 90 | 90 |
| 64 | 0*20+1*0+2*0+3*80 | 240 | 0*20+1*0+2*0+3*80 | 240 | 0*30+1*0+2*0+3*70 | 210 | 230 | 0*40+1*0+2*60+3*0 | 120 | 0*40+1*0+2*60+3*0 | 120 | 0*40+1*0+2*60+3*0 | 120 | 120 |
| 65 | 0*20+1*0+2*0+3*80 | 240 | 0*20+1*0+2*0+3*80 | 240 | 0*20+1*0+2*0+3*80 | 240 | 240 | 0*0+1*0+2*100+3*0 | 200 | 0*30+1*0+2*0+3*70 | 210 | 0*20+1*0+2*80+3*0 | 160 | 190 |
| 66 | 0*0+1*0+2*0+3*100 | 300 | 0*10+1*0+2*0+3*90 | 270 | 0*0+1*0+2*0+3*100 | 300 | 290 | 0*0+1*100+2*0+3*0 | 100 | 0*0+1*100+2*0+3*0 | 100 | 0*0+1*100+2*0+3*0 | 100 | 100 |
| 67 | 0*0+1*0+2*100+3*0 | 200 | 0*0+1*0+2*100+3*0 | 200 | 0*0+1*0+2*100+3*0 | 200 | 200 | 0*30+1*0+2*70+3*0 | 140 | 0*40+1*0+2*60+3*0 | 120 | 0*30+1*10+2*60+3*0 | 130 | 130 |
| 68 | 0*30+1*0+2*0+3*70 | 210 | 0*20+1*0+2*0+3*80 | 240 | 0*0+1*0+2*0+3*100 | 300 | 250 | 0*50+1*50+2*0+3*0 | 50 | 0*10+1*90+2*0+3*0 | 90 | 0*30+1*70+2*0+3*0 | 70 | 70 |
| 69 | 0*20+1*10+2*70+3*0 | 150 | 0*20+1*10+2*70+3*0 | 150 | 0*20+1*10+2*70+3*0 | 150 | 150 | 0*30+1*70+2*0+3*0 | 70 | 0*30+1*70+2*0+3*0 | 70 | 0*30+1*70+2*0+3*0 | 70 | 70 |
| 70 | 0*30+1*0+2*0+3*70 | 210 | 0*30+1*0+2*0+3*70 | 210 | 0*30+1*0+2*0+3*70 | 210 | 210 | 0*30+1*0+2*70+3*0 | 140 | 0*30+1*0+2*70+3*0 | 140 | 0*40+1*10+2*50+3*0 | 110 | 130 |
| 71 | 0*10+1*10+2*0+3*80 | 250 | 0*10+1*10+2*0+3*80 | 250 | 0*10+1*10+2*0+3*80 | 250 | 250 | 0*10+1*90+2*0+3*0 | 90 | 0*10+1*90+2*0+3*0 | 90 | 0*10+1*90+2*0+3*0 | 90 | 90 |
| 72 | 0*40+1*10+2*50+3*0 | 110 | 0*40+1*10+2*50+3*0 | 110 | 0*40+1*10+2*50+3*0 | 110 | 110 | 0*10+1*90+2*0+3*0 | 90 | 0*10+1*90+2*0+3*0 | 90 | 0*10+1*90+2*0+3*0 | 90 | 90 |
| 73 | 0*40+1*10+2*50+3*0 | 110 | 0*40+1*10+2*50+3*0 | 110 | 0*40+1*10+2*50+3*0 | 110 | 110 | 0*0+1*100+2*0+3*0 | 100 | 0*0+1*100+2*0+3*0 | 100 | 0*0+1*100+2*0+3*0 | 100 | 100 |
| 74 | 0*0+1*0+2*100+3*0 | 200 | 0*0+1*0+2*100+3*0 | 200 | 0*0+1*0+2*100+3*0 | 200 | 200 | 0*20+1*10+2*70+3*0 | 150 | 0*10+1*10+2*80+3*0 | 170 | 0*0+1*10+2*90+3*0 | 190 | 170 |
| 75 | 0*30+1*0+2*0+3*70 | 210 | 0*30+1*0+2*0+3*70 | 210 | 0*30+1*0+2*0+3*70 | 210 | 210 | 0*30+1*0+2*0+3*70 | 210 | 0*30+1*0+2*0+3*70 | 210 | 0*30+1*0+2*0+3*70 | 210 | 210 |
| 76 | 0*20+1*0+2*10+3*70 | 230 | 0*20+1*0+2*10+3*70 | 230 | 0*0+1*0+2*100+3*0 | 200 | 220 | 0*50+1*0+2*50+3*0 | 100 | 0*30+1*10+2*60+3*0 | 130 | 0*0+1*100+2*0+3*0 | 100 | 110 |
| 77 | 0*20+1*0+2*10+3*70 | 230 | 0*20+1*0+2*10+3*70 | 230 | 0*20+1*0+2*10+3*70 | 230 | 230 | 0*30+1*0+2*70+3*0 | 140 | 0*30+1*0+2*70+3*0 | 140 | 0*30+1*0+2*70+3*0 | 140 | 140 |
| 78 | 0*10+1*10+2*0+3*80 | 250 | 0*10+1*10+2*0+3*80 | 250 | 0*10+1*10+2*0+3*80 | 250 | 250 | 0*0+1*100+2*0+3*0 | 100 | 0*0+1*100+2*0+3*0 | 100 | 0*0+1*100+2*0+3*0 | 100 | 100 |
| 79 | 0*10+1*0+2*0+3*90 | 270 | 0*10+1*0+2*0+3*90 | 270 | 0*10+1*0+2*0+3*90 | 270 | 270 | 0*40+1*10+2*50+3*0 | 110 | 0*40+1*10+2*50+3*0 | 110 | 0*40+1*10+2*50+3*0 | 110 | 110 |
| 80 | 0*10+1*90+2*0+3*0 | 90 | 0*10+1*90+2*0+3*0 | 90 | 0*10+1*90+2*0+3*0 | 90 | 90 | 0*20+1*80+2*0+3*0 | 80 | 0*20+1*80+2*0+3*0 | 80 | 0*20+1*80+2*0+3*0 | 80 | 80 |
| 81 | 0*30+1*70+2*0+3*0 | 70 | 0*30+1*70+2*0+3*0 | 70 | 0*30+1*70+2*0+3*0 | 70 | 70 | 0*20+1*10+2*70+3*0 | 150 | 0*40+1*0+2*60+3*0 | 120 | 0*20+1*10+2*70+3*0 | 150 | 140 |
| 82 | 0*20+1*0+2*80+3*0 | 160 | 0*20+1*0+2*80+3*0 | 160 | 0*20+1*0+2*80+3*0 | 160 | 160 | 0*10+1*90+2*0+3*0 | 90 | 0*10+1*90+2*0+3*0 | 90 | 0*10+1*90+2*0+3*0 | 90 | 90 |
| 83 | 0*0+1*0+2*100+3*0 | 200 | 0*0+1*0+2*100+3*0 | 200 | 0*0+1*0+2*100+3*0 | 200 | 200 | 0*20+1*10+2*70+3*0 | 150 | 0*20+1*10+2*70+3*0 | 150 | 0*20+1*10+2*70+3*0 | 150 | 150 |
| 84 | 0*30+1*0+2*0+3*70 | 210 | 0*40+1*0+2*60+3*0 | 210 | 0*20+1*0+2*0+3*80 | 240 | 220 | 0*0+1*100+2*0+3*0 | 100 | 0*0+1*100+2*0+3*0 | 100 | 0*0+1*100+2*0+3*0 | 100 | 100 |
| 85 | 0*60+1*40+2*0+3*0 | 40 | 0*60+1*40+2*0+3*0 | 40 | 0*60+1*40+2*0+3*0 | 40 | 40 | 0*40+1*0+2*60+3*0 | 210 | 0*40+1*0+2*60+3*0 | 210 | 0*40+1*0+2*60+3*0 | 210 | 210 |
| 86 | 0*40+1*10+2*50+3*0 | 110 | 0*30+1*10+2*60+3*0 | 130 | 0*20+1*10+2*70+3*0 | 150 | 130 | 0*40+1*0+2*60+3*0 | 120 | 0*40+1*0+2*60+3*0 | 120 | 0*40+1*0+2*60+3*0 | 120 | 120 |
| 87 | 0*30+1*0+2*0+3*70 | 210 | 0*30+1*0+2*0+3*70 | 210 | 0*30+1*0+2*0+3*70 | 210 | 210 | 0*10+1*90+2*0+3*0 | 90 | 0*10+1*90+2*0+3*0 | 90 | 0*10+1*90+2*0+3*0 | 90 | 90 |
| 88 | 0*20+1*0+2*80+3*0 | 160 | 0*20+1*0+2*80+3*0 | 160 | 0*0+1*10+2*90+3*0 | 190 | 170 | 0*20+1*0+2*80+3*0 | 160 | 0*30+1*0+2*70+3*0 | 140 | 0*20+1*0+2*80+3*0 | 180 | 160 |
| 89 | 0*10+1*0+2*0+3*90 | 270 | 0*20+1*0+2*0+3*80 | 240 | 0*10+1*0+2*0+3*90 | 270 | 260 | 0*10+1*0+2*90+3*0 | 180 | 0*10+1*0+2*90+3*0 | 180 | 0*10+1*0+2*90+3*0 | 180 | 180 |
| 90 | 0*30+1*0+2*70+3*0 | 140 | 0*30+1*0+2*70+3*0 | 140 | 0*30+1*0+2*70+3*0 | 140 | 140 | 0*10+1*0+2*90+3*0 | 180 | 0*10+1*0+2*90+3*0 | 180 | 0*10+1*0+2*90+3*0 | 180 | 180 |
| 91 | 0*0+1*0+2*100+3*0 | 200 | 0*30+1*0+2*0+3*70 | 210 | 0*20+1*0+2*80+3*0 | 160 | 190 | 0*10+1*90+2*0+3*0 | 90 | 0*10+1*90+2*0+3*0 | 90 | 0*10+1*90+2*0+3*0 | 90 | 90 |
| 92 | 0*0+1*0+2*100+3*0 | 200 | 0*0+1*0+2*100+3*0 | 200 | 0*0+1*0+2*100+3*0 | 200 | 200 | 0*10+1*0+2*0+3*90 | 270 | 0*20+1*0+2*0+3*80 | 240 | 0*20+1*0+2*0+3*80 | 240 | 250 |
| 93 | 0*30+1*0+2*70+3*0 | 140 | 0*20+1*10+2*70+3*0 | 150 | 0*50+1*0+2*50+3*0 | 100 | 130 | 0*0+1*100+2*0+3*0 | 100 | 0*40+1*0+2*60+3*0 | 120 | 0*40+1*10+2*50+3*0 | 110 | 110 |
| 94 | 0*40+1*0+2*0+3*60 | 180 | 0*20+1*0+2*80+3*0 | 160 | 0*10+1*10+2*80+3*0 | 170 | 170 | 0*40+1*0+2*0+3*60 | 180 | 0*20+1*0+2*80+3*0 | 160 | 0*10+1*10+2*80+3*0 | 170 | 170 |
| 95 | 0*20+1*0+2*0+3*80 | 240 | 0*20+1*0+2*0+3*80 | 240 | 0*20+1*0+2*0+3*80 | 240 | 240 | 0*10+1*90+2*0+3*0 | 90 | 0*10+1*90+2*0+3*0 | 90 | 0*10+1*90+2*0+3*0 | 90 | 90 |
| 96 | 0*0+1*0+2*0+3*100 | 300 | 0*0+1*0+2*0+3*100 | 300 | 0*0+1*0+2*0+3*100 | 300 | 300 | 0*40+1*60+2*0+3*0 | 60 | 0*20+1*80+2*0+3*0 | 80 | 0*30+1*70+2*0+3*0 | 70 | 70 |
| 97 | 0*20+1*10+2*70+3*0 | 150 | 0*40+1*0+2*60+3*0 | 120 | 0*40+1*0+2*60+3*0 | 120 | 130 | 0*20+1*80+2*0+3*0 | 80 | 0*20+1*80+2*0+3*0 | 80 | 0*20+1*80+2*0+3*0 | 80 | 80 |
| 98 | 0*30+1*0+2*0+3*70 | 210 | 0*30+1*0+2*0+3*70 | 210 | 0*30+1*0+2*0+3*70 | 210 | 210 | 0*0+1*100+2*0+3*0 | 100 | 0*0+1*100+2*0+3*0 | 100 | 0*0+1*100+2*0+3*0 | 100 | 100 |
| 99 | 0*0+1*0+2*100+3*0 | 200 | 0*0+1*0+2*100+3*0 | 200 | 0*0+1*0+2*100+3*0 | 200 | 200 | 0*20+1*80+2*0+3*0 | 80 | 0*20+1*80+2*0+3*0 | 80 | 0*20+1*80+2*0+3*0 | 80 | 80 |
| 100 | 0*20+1*0+2*0+3*80 | 240 | 0*30+1*0+2*0+3*70 | 210 | 0*30+1*0+2*0+3*70 | 210 | 220 | 0*0+1*100+2*0+3*0 | 100 | 0*0+1*100+2*0+3*0 | 100 | 0*0+1*100+2*0+3*0 | 100 | 100 |
| 101 | 0*40+1*10+2*0+3*50 | 160 | 0*0+1*70+2*30+3*0 | 130 | 0*0+1*70+2*30+3*0 | 130 | 140 | 0*0+1*100+2*0+3*0 | 100 | 0*0+1*100+2*0+3*0 | 100 | 0*0+1*100+2*0+3*0 | 100 | 100 |
| 102 | 0*30+1*0+2*70+3*0 | 140 | 0*30+1*0+2*70+3*0 | 140 | 0*30+1*0+2*70+3*0 | 140 | 140 | 0*0+1*100+2*0+3*0 | 100 | 0*0+1*100+2*0+3*0 | 100 | 0*0+1*100+2*0+3*0 | 100 | 100 |
| 103 | 0*20+1*0+2*0+3*80 | 240 | 0*0+1*0+2*100+3*0 | 200 | 0*30+1*10+2*0+3*60 | 190 | 210 | 0*50+1*50+2*0+3*0 | 50 | 0*0+1*90+2*0+3*0 | 90 | 0*0+1*100+2*0+3*0 | 100 | 80 |
| 104 | 0*0+1*0+2*100+3*0 | 200 | 0*0+1*40+2*0+3*60 | 220 | 0*20+1*10+2*70+3*0 | 150 | 190 | 0*30+1*70+2*0+3*0 | 70 | 0*50+1*0+2*50+3*0 | 100 | 0*50+1*0+2*50+3*0 | 100 | 90 |
| 105 | 0*20+1*0+2*80+3*0 | 160 | 0*20+1*0+2*80+3*0 | 160 | 0*20+1*0+2*80+3*0 | 160 | 160 | 0*20+1*10+2*70+3*0 | 150 | 0*20+1*10+2*70+3*0 | 150 | 0*20+1*10+2*70+3*0 | 150 | 150 |
| 106 | 0*50+1*0+2*10+3*40 | 140 | 0*50+1*0+2*10+3*40 | 140 | 0*50+1*0+2*10+3*40 | 140 | 140 | 0*0+1*0+2*100+3*0 | 200 | 0*20+1*0+2*80+3*0 | 160 | 0*20+1*10+2*70+3*0 | 150 | 170 |
| 107 | 0*30+1*0+2*0+3*70 | 210 | 0*30+1*0+2*0+3*70 | 210 | 0*30+1*0+2*0+3*70 | 210 | 210 | 0*20+1*0+2*80+3*0 | 160 | 0*20+1*0+2*80+3*0 | 160 | 0*20+1*0+2*80+3*0 | 160 | 160 |
| 108 | 0*20+1*80+2*0+3*0 | 80 | 0*20+1*80+2*0+3*0 | 80 | 0*20+1*80+2*0+3*0 | 80 | 80 | 0*50+1*50+2*0+3*0 | 50 | 0*50+1*50+2*0+3*0 | 50 | 0*50+1*50+2*0+3*0 | 50 | 50 |
| 109 | 0*0+1*0+2*50+3*50 | 250 | 0*0+1*0+2*50+3*50 | 250 | 0*0+1*0+2*50+3*50 | 250 | 250 | 0*30+1*0+2*70+3*0 | 140 | 0*30+1*0+2*70+3*0 | 140 | 0*30+1*0+2*70+3*0 | 140 | 140 |
| 110 | 0*0+1*0+2*0+3*100 | 300 | 0*0+1*0+2*0+3*100 | 300 | 0*0+1*0+2*0+3*100 | 300 | 300 | 0*0+1*100+2*0+3*0 | 100 | 0*40+1*10+2*50+3*0 | 110 | 0*40+1*0+2*60+3*0 | 120 | 110 |
| 111 | 0*10+1*90+2*0+3*0 | 90 | 0*10+1*90+2*0+3*0 | 90 | 0*10+1*90+2*0+3*0 | 90 | 90 | 0*10+1*10+2*0+3*80 | 250 | 0*10+1*10+2*0+3*80 | 250 | 0*10+1*10+2*0+3*80 | 250 | 250 |
| 112 | 0*10+1*10+2*0+3*80 | 250 | 0*10+1*10+2*0+3*80 | 250 | 0*10+1*10+2*0+3*80 | 250 | 250 | 0*60+1*0+2*40+3*0 | 80 | 0*60+1*0+2*40+3*0 | 80 | 0*60+1*0+2*40+3*0 | 80 | 80 |
| 113 | 0*30+1*0+2*0+3*70 | 210 | 0*40+1*0+2*10+3*50 | 170 | 0*30+1*10+2*0+3*60 | 190 | 190 | 0*50+1*0+2*50+3*0 | 100 | 0*50+1*0+2*50+3*0 | 100 | 0*50+1*0+2*50+3*0 | 100 | 100 |
| 114 | 0*10+1*0+2*0+3*90 | 270 | 0*10+1*0+2*0+3*90 | 270 | 0*10+1*0+2*0+3*90 | 270 | 270 | 0*10+1*90+2*0+3*0 | 90 | 0*10+1*90+2*0+3*0 | 90 | 0*10+1*90+2*0+3*0 | 90 | 90 |
| 115 | 0*10+1*0+2*0+3*90 | 270 | 0*10+1*0+2*0+3*90 | 270 | 0*10+1*0+2*0+3*90 | 270 | 270 | 0*30+1*0+2*70+3*0 | 140 | 0*30+1*0+2*70+3*0 | 140 | 0*30+1*0+2*70+3*0 | 140 | 140 |
| 116 | 0*0+1*0+2*0+3*100 | 300 | 0*0+1*0+2*0+3*100 | 300 | 0*0+1*0+2*0+3*100 | 300 | 300 | 0*10+1*0+2*90+3*0 | 180 | 0*10+1*0+2*90+3*0 | 180 | 0*10+1*0+2*90+3*0 | 180 | 180 |
| 117 | 0*30+1*0+2*10+3*60 | 200 | 0*20+1*0+2*10+3*70 | 230 | 0*10+1*0+2*10+3*80 | 260 | 230 | 0*30+1*10+2*60+3*0 | 130 | 0*30+1*10+2*60+3*0 | 130 | 0*30+1*10+2*60+3*0 | 160 | 140 |
| 118 | 0*30+1*0+2*0+3*70 | 210 | 0*40+1*0+2*0+3*60 | 180 | 0*40+1*0+2*0+3*60 | 180 | 190 | 0*10+1*0+2*90+3*0 | 180 | 0*10+1*0+2*90+3*0 | 180 | 0*10+1*0+2*90+3*0 | 180 | 180 |
| 119 | 0*10+1*0+2*0+3*90 | 270 | 0*10+1*0+2*0+3*90 | 270 | 0*10+1*0+2*0+3*90 | 270 | 270 | 0*0+1*70+2*30+3*0 | 130 | 0*0+1*70+2*30+3*0 | 130 | 0*0+1*70+2*30+3*0 | 130 | 130 |
| 120 | 0*20+1*0+2*10+3*70 | 230 | 0*20+1*0+2*10+3*70 | 230 | 0*20+1*0+2*10+3*70 | 230 | 230 | 0*50+1*50+2*0+3*0 | 50 | 0*50+1*50+2*0+3*0 | 50 | 0*50+1*50+2*0+3*0 | 50 | 50 |
| 121 | 0*0+1*0+2*100+3*0 | 200 | 0*0+1*0+2*100+3*0 | 200 | 0*0+1*0+2*100+3*0 | 200 | 200 | 0*40+1*0+2*60+3*0 | 120 | 0*30+1*0+2*70+3*0 | 140 | 0*20+1*0+2*80+3*0 | 160 | 140 |
| 122 | 0*0+1*0+2*100+3*0 | 200 | 0*10+1*10+2*0+3*80 | 250 | 0*20+1*0+2*0+3*80 | 240 | 230 | 0*0+1*0+2*100+3*0 | 200 | 0*10+1*0+2*90+3*0 | 180 | 0*0+1*10+2*90+3*0 | 190 | 190 |
| 123 | 0*0+1*40+2*0+3*60 | 220 | 0*20+1*10+2*0+3*70 | 220 | 0*20+1*10+2*0+3*70 | 220 | 220 | 0*20+1*80+2*0+3*0 | 80 | 0*20+1*80+2*0+3*0 | 80 | 0*20+1*80+2*0+3*0 | 80 | 80 |
| 124 | 0*0+1*0+2*0+3*100 | 300 | 0*10+1*0+2*0+3*90 | 270 | 0*10+1*0+2*0+3*90 | 270 | 280 | 0*20+1*10+2*70+3*0 | 150 | 0*20+1*10+2*70+3*0 | 150 | 0*20+1*10+2*70+3*0 | 150 | 150 |
| 125 | 0*10+1*10+2*0+3*80 | 250 | 0*10+1*10+2*0+3*80 | 250 | 0*10+1*10+2*0+3*80 | 250 | 250 | 0*10+1*0+2*90+3*0 | 180 | 0*10+1*0+2*90+3*0 | 180 | 0*10+1*0+2*90+3*0 | 180 | 180 |
| 126 | 0*20+1*0+2*0+3*80 | 240 | 0*20+1*0+2*0+3*80 | 240 | 0*20+1*0+2*0+3*80 | 240 | 240 | 0*10+1*0+2*90+3*0 | 180 | 0*10+1*0+2*90+3*0 | 180 | 0*10+1*0+2*90+3*0 | 180 | 180 |
| 127 | 0*0+1*0+2*0+3*100 | 300 | 0*10+1*0+2*0+3*90 | 270 | 0*0+1*0+2*0+3*100 | 300 | 290 | 0*0+1*100+2*0+3*0 | 100 | 0*0+1*100+2*0+3*0 | 100 | 0*0+1*100+2*0+3*0 | 100 | 100 |
| 128 | 0*20+1*0+2*10+3*70 | 230 | 0*20+1*0+2*10+3*70 | 230 | 0*20+1*0+2*10+3*70 | 230 | 230 | 0*10+1*0+2*90+3*0 | 180 | 0*10+1*0+2*90+3*0 | 180 | 0*10+1*0+2*90+3*0 | 180 | 180 |
| 129 | 0*10+1*10+2*0+3*80 | 250 | 0*10+1*10+2*0+3*80 | 250 | 0*10+1*10+2*0+3*80 | 250 | 250 | 0*30+1*10+2*60+3*0 | 130 | 0*30+1*10+2*60+3*0 | 130 | 0*30+1*10+2*60+3*0 | 130 | 130 |
| 130 | 0*0+1*10+2*90+3*0 | 190 | 0*0+1*10+2*90+3*0 | 190 | 0*0+1*10+2*90+3*0 | 190 | 190 | 0*10+1*0+2*90+3*0 | 180 | 0*10+1*0+2*90+3*0 | 180 | 0*10+1*0+2*90+3*0 | 180 | 180 |
| 131 | 0*20+1*0+2*0+3*80 | 240 | 0*20+1*0+2*0+3*80 | 240 | 0*20+1*0+2*0+3*80 | 240 | 240 | 0*0+1*10+2*90+3*0 | 190 | 0*0+1*10+2*90+3*0 | 190 | 0*0+1*10+2*90+3*0 | 190 | 190 |
| 132 | 0*20+1*0+2*10+3*70 | 230 | 0*20+1*0+2*10+3*70 | 230 | 0*20+1*0+2*10+3*70 | 230 | 230 | 0*10+1*90+2*0+3*0 | 90 | 0*10+1*90+2*0+3*0 | 90 | 0*10+1*90+2*0+3*0 | 90 | 90 |
| 133 | 0*50+1*50+2*0+3*0 | 50 | 0*50+1*50+2*0+3*0 | 50 | 0*50+1*50+2*0+3*0 | 50 | 50 | 0*0+1*10+2*90+3*0 | 190 | 0*0+1*10+2*90+3*0 | 190 | 0*0+1*10+2*90+3*0 | 190 | 190 |
| 134 | 0*10+1*10+2*0+3*80 | 250 | 0*0+1*40+2*0+3*60 | 220 | 0*10+1*10+2*0+3*80 | 250 | 240 | 0*10+1*0+2*90+3*0 | 180 | 0*0+1*0+2*100+3*0 | 200 | 0*20+1*0+2*80+3*0 | 160 | 180 |
| 135 | 0*0+1*0+2*100+3*0 | 200 | 0*20+1*0+2*0+3*80 | 240 | 0*20+1*10+2*0+3*70 | 220 | 220 | 0*20+1*10+2*70+3*0 | 150 | 0*20+1*10+2*70+3*0 | 150 | 0*20+1*10+2*70+3*0 | 150 | 150 |
| 136 | 0*40+1*10+2*50+3*0 | 110 | 0*40+1*10+2*50+3*0 | 110 | 0*40+1*10+2*50+3*0 | 110 | 110 | 0*100+1*0+2*0+3*0 | 0 | 0*100+1*0+2*0+3*0 | 0 | 0*100+1*0+2*0+3*0 | 0 | 0 |
| 137 | 0*20+1*10+2*0+3*70 | 220 | 0*20+1*10+2*0+3*70 | 220 | 0*20+1*10+2*0+3*70 | 220 | 220 | 0*0+1*100+2*0+3*0 | 100 | 0*0+1*100+2*0+3*0 | 100 | 0*0+1*100+2*0+3*0 | 100 | 100 |
| 138 | 0*30+1*0+2*0+3*70 | 210 | 0*30+1*0+2*0+3*70 | 210 | 0*30+1*0+2*0+3*70 | 210 | 210 | 0*10+1*0+2*90+3*0 | 180 | 0*20+1*10+2*70+3*0 | 150 | 0*10+1*0+2*90+3*0 | 180 | 170 |
| 139 | 0*20+1*0+2*80+3*0 | 160 | 0*20+1*0+2*80+3*0 | 160 | 0*20+1*0+2*80+3*0 | 160 | 160 | 0*30+1*0+2*0+3*70 | 210 | 0*30+1*0+2*0+3*70 | 210 | 0*30+1*0+2*0+3*70 | 210 | 210 |
| 140 | 0*10+1*90+2*0+3*0 | 90 | 0*10+1*90+2*0+3*0 | 90 | 0*10+1*90+2*0+3*0 | 90 | 90 | 0*0+1*0+2*100+3*0 | 200 | 0*10+1*10+2*80+3*0 | 170 | 0*0+1*0+2*100+3*0 | 200 | 190 |
| 141 | 0*20+1*0+2*10+3*70 | 230 | 0*20+1*0+2*10+3*70 | 230 | 0*20+1*0+2*10+3*70 | 230 | 230 | 0*30+1*0+2*70+3*0 | 140 | 0*30+1*0+2*70+3*0 | 140 | 0*30+1*0+2*70+3*0 | 140 | 140 |
| 142 | 0*0+1*10+2*0+3*90 | 280 | 0*10+1*10+2*0+3*80 | 250 | 0*10+1*10+2*0+3*80 | 250 | 260 | 0*60+1*40+2*0+3*0 | 40 | 0*60+1*40+2*0+3*0 | 40 | 0*60+1*40+2*0+3*0 | 40 | 40 |
| 143 | 0*40+1*0+2*60+3*0 | 120 | 0*40+1*0+2*60+3*0 | 120 | 0*40+1*0+2*60+3*0 | 120 | 120 | 0*40+1*60+2*0+3*0 | 60 | 0*40+1*60+2*0+3*0 | 60 | 0*40+1*60+2*0+3*0 | 60 | 60 |
| 144 | 0*0+1*100+2*0+3*0 | 100 | 0*30+1*10+2*60+3*0 | 130 | 0*0+1*100+2*0+3*0 | 100 | 110 | 0*20+1*80+2*0+3*0 | 80 | 0*10+1*90+2*0+3*0 | 90 | 0*0+1*100+2*0+3*0 | 100 | 90 |
| 145 | 0*10+1*0+2*90+3*0 | 180 | 0*10+1*0+2*90+3*0 | 180 | 0*10+1*0+2*90+3*0 | 180 | 180 | 0*10+1*10+2*80+3*0 | 170 | 0*10+1*10+2*80+3*0 | 170 | 0*10+1*10+2*80+3*0 | 170 | 170 |
| 146 | 0*20+1*0+2*0+3*80 | 240 | 0*20+1*0+2*0+3*80 | 240 | 0*20+1*0+2*0+3*80 | 240 | 240 | 0*30+1*10+2*60+3*0 | 130 | 0*30+1*10+2*60+3*0 | 130 | 0*30+1*10+2*60+3*0 | 130 | 130 |
| 147 | 0*10+1*10+2*0+3*80 | 250 | 0*20+1*10+2*0+3*70 | 220 | 0*10+1*10+2*0+3*80 | 250 | 240 | 0*10+1*90+2*0+3*0 | 90 | 0*10+1*90+2*0+3*0 | 90 | 0*10+1*90+2*0+3*0 | 90 | 90 |
| 148 | 0*40+1*0+2*0+3*60 | 180 | 0*40+1*0+2*0+3*60 | 180 | 0*40+1*0+2*0+3*60 | 180 | 180 | 0*0+1*0+2*100+3*0 | 200 | 0*20+1*0+2*80+3*0 | 160 | 0*20+1*10+2*70+3*0 | 150 | 170 |
| 149 | 0*20+1*0+2*10+3*70 | 230 | 0*20+1*0+2*10+3*70 | 230 | 0*20+1*0+2*10+3*70 | 230 | 230 | 0*60+1*0+2*40+3*0 | 80 | 0*60+1*0+2*40+3*0 | 80 | 0*60+1*0+2*40+3*0 | 80 | 80 |
| 150 | 0*20+1*0+2*80+3*0 | 160 | 0*20+1*0+2*80+3*0 | 160 | 0*20+1*0+2*80+3*0 | 160 | 160 | 0*10+1*90+2*0+3*0 | 90 | 0*10+1*90+2*0+3*0 | 90 | 0*10+1*90+2*0+3*0 | 90 | 90 |
| 151 | 0*50+1*0+2*0+3*50 | 150 | 0*50+1*0+2*0+3*50 | 150 | 0*50+1*0+2*0+3*50 | 150 | 150 | 0*40+1*0+2*60+3*0 | 120 | 0*40+1*0+2*60+3*0 | 120 | 0*40+1*0+2*60+3*0 | 120 | 120 |
| 152 | 0*0+1*0+2*0+3*100 | 300 | 0*0+1*0+2*0+3*100 | 300 | 0*0+1*0+2*0+3*100 | 300 | 300 | 0*20+1*10+2*70+3*0 | 150 | 0*20+1*10+2*70+3*0 | 150 | 0*20+1*10+2*70+3*0 | 150 | 150 |
| 153 | 0*20+1*0+2*0+3*80 | 240 | 0*30+1*0+2*0+3*70 | 210 | 0*30+1*0+2*0+3*70 | 210 | 220 | 0*20+1*0+2*80+3*0 | 160 | 0*20+1*0+2*80+3*0 | 160 | 0*20+1*0+2*80+3*0 | 160 | 160 |
| 154 | 0*0+1*40+2*0+3*60 | 220 | 0*0+1*40+2*0+3*60 | 220 | 0*0+1*40+2*0+3*60 | 220 | 220 | 0*70+1*30+2*0+3*0 | 30 | 0*70+1*30+2*0+3*0 | 30 | 0*70+1*30+2*0+3*0 | 30 | 30 |
| 155 | 0*20+1*0+2*0+3*80 | 240 | 0*20+1*0+2*0+3*80 | 240 | 0*20+1*0+2*0+3*80 | 240 | 240 | 0*0+1*100+2*0+3*0 | 100 | 0*0+1*100+2*0+3*0 | 100 | 0*0+1*100+2*0+3*0 | 100 | 100 |
| 156 | 0*30+1*0+2*70+3*0 | 140 | 0*30+1*0+2*70+3*0 | 140 | 0*30+1*0+2*70+3*0 | 140 | 140 | 0*20+1*0+2*80+3*0 | 160 | 0*20+1*0+2*80+3*0 | 160 | 0*20+1*0+2*80+3*0 | 160 | 160 |
| 157 | 0*10+1*0+2*0+3*90 | 270 | 0*10+1*0+2*0+3*90 | 270 | 0*10+1*0+2*0+3*90 | 270 | 270 | 0*30+1*10+2*60+3*0 | 130 | 0*30+1*10+2*60+3*0 | 130 | 0*30+1*10+2*60+3*0 | 130 | 130 |
| 158 | 0*10+1*10+2*0+3*80 | 250 | 0*10+1*10+2*0+3*80 | 250 | 0*10+1*10+2*0+3*80 | 250 | 250 | 0*10+1*90+2*0+3*0 | 90 | 0*10+1*90+2*0+3*0 | 90 | 0*10+1*90+2*0+3*0 | 90 | 90 |
| 159 | 0*20+1*10+2*0+3*70 | 220 | 0*20+1*10+2*0+3*70 | 220 | 0*20+1*10+2*0+3*70 | 220 | 220 | 0*50+1*0+2*0+3*50 | 150 | 0*50+1*0+2*0+3*50 | 150 | 0*50+1*0+2*0+3*50 | 150 | 150 |
| 160 | 0*40+1*0+2*60+3*0 | 120 | 0*40+1*0+2*60+3*0 | 120 | 0*40+1*0+2*60+3*0 | 120 | 120 | 0*40+1*0+2*0+3*60 | 180 | 0*40+1*0+2*0+3*60 | 180 | 0*40+1*0+2*0+3*60 | 180 | 180 |
| 161 | 0*30+1*0+2*0+3*70 | 210 | 0*20+1*0+2*0+3*80 | 240 | 0*20+1*0+2*0+3*80 | 240 | 230 | 0*0+1*100+2*0+3*0 | 100 | 0*0+1*100+2*0+3*0 | 100 | 0*0+1*100+2*0+3*0 | 100 | 100 |
| 162 | 0*50+1*0+2*0+3*50 | 150 | 0*50+1*0+2*0+3*50 | 150 | 0*50+1*0+2*0+3*50 | 150 | 150 | 0*10+1*90+2*0+3*0 | 90 | 0*10+1*90+2*0+3*0 | 90 | 0*10+1*90+2*0+3*0 | 90 | 90 |
| 163 | 0*20+1*0+2*0+3*80 | 240 | 0*0+1*0+2*100+3*0 | 200 | 0*0+1*40+2*0+3*60 | 220 | 220 | 0*30+1*70+2*0+3*0 | 70 | 0*30+1*70+2*0+3*0 | 70 | 0*30+1*70+2*0+3*0 | 70 | 70 |
| 164 | 0*10+1*10+2*0+3*80 | 250 | 0*10+1*10+2*0+3*80 | 250 | 0*10+1*10+2*0+3*80 | 250 | 250 | 0*10+1*90+2*0+3*0 | 90 | 0*10+1*90+2*0+3*0 | 90 | 0*10+1*90+2*0+3*0 | 90 | 90 |
| 165 | 0*10+1*90+2*0+3*0 | 90 | 0*10+1*90+2*0+3*0 | 90 | 0*10+1*90+2*0+3*0 | 90 | 90 | 0*20+1*10+2*70+3*0 | 150 | 0*20+1*10+2*70+3*0 | 150 | 0*20+1*10+2*70+3*0 | 150 | 150 |
| 166 | 0*50+1*0+2*0+3*50 | 150 | 0*60+1*0+2*0+3*40 | 120 | 0*60+1*0+2*0+3*40 | 120 | 130 | 0*10+1*0+2*90+3*0 | 180 | 0*10+1*0+2*90+3*0 | 180 | 0*10+1*0+2*90+3*0 | 180 | 180 |
| 167 | 0*20+1*10+2*0+3*70 | 220 | 0*20+1*10+2*0+3*70 | 220 | 0*20+1*10+2*0+3*70 | 220 | 220 | 0*10+1*90+2*0+3*0 | 90 | 0*10+1*90+2*0+3*0 | 90 | 0*10+1*90+2*0+3*0 | 90 | 90 |
| 168 | 0*20+1*0+2*10+3*70 | 230 | 0*20+1*0+2*10+3*70 | 230 | 0*20+1*0+2*10+3*70 | 230 | 230 | 0*0+1*70+2*30+3*0 | 130 | 0*0+1*70+2*30+3*0 | 130 | 0*0+1*70+2*30+3*0 | 130 | 130 |
| 169 | 0*50+1*0+2*10+3*40 | 140 | 0*50+1*0+2*10+3*40 | 140 | 0*50+1*0+2*10+3*40 | 140 | 140 | 0*10+1*90+2*0+3*0 | 90 | 0*10+1*90+2*0+3*0 | 90 | 0*10+1*90+2*0+3*0 | 90 | 90 |
| 170 | 0*0+1*10+2*0+3*90 | 280 | 0*0+1*20+2*0+3*80 | 260 | 0*20+1*0+2*0+3*80 | 240 | 260 | 0*50+1*0+2*0+3*50 | 150 | 0*50+1*0+2*0+3*50 | 150 | 0*50+1*0+2*0+3*50 | 150 | 150 |
| 171 | 0*20+1*0+2*0+3*80 | 240 | 0*20+1*0+2*0+3*80 | 240 | 0*20+1*0+2*0+3*80 | 240 | 240 | 0*50+1*0+2*50+3*0 | 100 | 0*50+1*0+2*50+3*0 | 100 | 0*50+1*0+2*50+3*0 | 100 | 100 |
| 172 | 0*20+1*0+2*0+3*80 | 240 | 0*30+1*0+2*0+3*70 | 210 | 0*30+1*0+2*0+3*70 | 210 | 220 | 0*50+1*0+2*0+3*50 | 150 | 0*50+1*0+2*0+3*50 | 150 | 0*50+1*0+2*0+3*50 | 150 | 150 |
| 173 | 0*20+1*10+2*0+3*70 | 220 | 0*20+1*10+2*0+3*70 | 220 | 0*20+1*10+2*0+3*70 | 220 | 220 | 0*0+1*40+2*0+3*60 | 220 | 0*0+1*40+2*0+3*60 | 220 | 0*0+1*40+2*0+3*60 | 220 | 220 |
| 174 | 0*70+1*30+2*0+3*0 | 30 | 0*70+1*30+2*0+3*0 | 30 | 0*40+1*60+2*0+3*0 | 60 | 40 | 0*0+1*50+2*50+3*0 | 150 | 0*0+1*50+2*50+3*0 | 150 | 0*0+1*50+2*50+3*0 | 150 | 150 |
| 175 | 0*0+1*40+2*0+3*60 | 220 | 0*0+1*40+2*0+3*60 | 220 | 0*0+1*40+2*0+3*60 | 220 | 220 | 0*0+1*70+2*30+3*0 | 130 | 0*0+1*70+2*30+3*0 | 130 | 0*0+1*70+2*30+3*0 | 130 | 130 |
| 176 | 0*0+1*20+2*0+3*80 | 260 | 0*20+1*0+2*10+3*70 | 230 | 0*20+1*0+2*10+3*70 | 230 | 240 | 0*40+1*60+2*0+3*0 | 60 | 0*40+1*60+2*0+3*0 | 60 | 0*40+1*60+2*0+3*0 | 60 | 60 |
| 177 | 0*20+1*0+2*0+3*80 | 240 | 0*20+1*0+2*0+3*80 | 240 | 0*20+1*0+2*0+3*80 | 240 | 240 | 0*30+1*0+2*0+3*70 | 210 | 0*10+1*0+2*90+3*0 | 180 | 0*40+1*0+2*0+3*60 | 180 | 190 |
| 178 | 0*10+1*0+2*0+3*90 | 270 | 0*10+1*0+2*0+3*90 | 270 | 0*10+1*0+2*0+3*90 | 270 | 270 | 0*0+1*40+2*0+3*60 | 220 | 0*0+1*40+2*0+3*60 | 220 | 0*0+1*40+2*0+3*60 | 220 | 220 |
| 179 | 0*20+1*0+2*0+3*80 | 240 | 0*20+1*0+2*0+3*80 | 240 | 0*20+1*0+2*0+3*80 | 240 | 240 | 0*0+1*70+2*30+3*0 | 130 | 0*0+1*70+2*30+3*0 | 130 | 0*0+1*70+2*30+3*0 | 130 | 130 |
| 180 | 0*0+1*0+2*100+3*0 | 200 | 0*10+1*10+2*80+3*0 | 170 | 0*10+1*10+2*80+3*0 | 170 | 180 | 0*20+1*80+2*0+3*0 | 80 | 0*20+1*80+2*0+3*0 | 80 | 0*20+1*80+2*0+3*0 | 80 | 80 |
| 181 | 0*0+1*0+2*100+3*0 | 200 | 0*0+1*0+2*100+3*0 | 200 | 0*0+1*0+2*100+3*0 | 200 | 200 | 0*10+1*0+2*40+3*50 | 230 | 0*30+1*0+2*0+3*70 | 210 | 0*0+1*40+2*0+3*60 | 220 | 220 |
| 182 | 0*20+1*0+2*80+3*0 | 160 | 0*20+1*0+2*80+3*0 | 160 | 0*20+1*0+2*80+3*0 | 160 | 160 | 0*20+1*0+2*80+3*0 | 160 | 0*0+1*30+2*70+3*0 | 170 | 0*10+1*0+2*90+3*0 | 180 | 170 |
| 183 | 0*10+1*10+2*0+3*80 | 250 | 0*0+1*20+2*0+3*80 | 260 | 0*10+1*0+2*0+3*90 | 270 | 260 | 0*20+1*80+2*0+3*0 | 80 | 0*10+1*90+2*0+3*0 | 90 | 0*50+1*0+2*50+3*0 | 100 | 90 |
| 184 | 0*20+1*0+2*0+3*80 | 240 | 0*20+1*0+2*10+3*70 | 230 | 0*20+1*0+2*0+3*80 | 250 | 240 | 0*30+1*70+2*0+3*0 | 70 | 0*20+1*80+2*0+3*0 | 80 | 0*10+1*90+2*0+3*0 | 90 | 80 |
| 185 | 0*30+1*0+2*0+3*70 | 210 | 0*0+1*40+2*0+3*60 | 220 | 0*20+1*0+2*10+3*70 | 230 | 220 | 0*20+1*80+2*0+3*0 | 80 | 0*20+1*80+2*0+3*0 | 80 | 0*20+1*80+2*0+3*0 | 80 | 80 |
| 186 | 0*40+1*10+2*50+3*0 | 110 | 0*40+1*10+2*50+3*0 | 110 | 0*40+1*10+2*50+3*0 | 110 | 110 | 0*20+1*0+2*80+3*0 | 160 | 0*20+1*0+2*80+3*0 | 160 | 0*20+1*0+2*80+3*0 | 160 | 160 |
| 187 | 0*60+1*0+2*0+3*40 | 120 | 0*60+1*0+2*0+3*40 | 120 | 0*60+1*0+2*0+3*40 | 120 | 120 | 0*0+1*40+2*0+3*60 | 220 | 0*0+1*0+2*70+3*30 | 230 | 0*20+1*0+2*0+3*80 | 240 | 230 |
| 188 | 0*0+1*30+2*70+3*0 | 170 | 0*0+1*30+2*70+3*0 | 170 | 0*0+1*30+2*70+3*0 | 170 | 170 | 0*20+1*80+2*0+3*0 | 80 | 0*20+1*80+2*0+3*0 | 80 | 0*20+1*80+2*0+3*0 | 80 | 80 |
| 189 | 0*20+1*0+2*0+3*80 | 240 | 0*20+1*0+2*0+3*80 | 240 | 0*20+1*0+2*0+3*80 | 240 | 240 | 0*50+1*50+2*0+3*0 | 50 | 0*50+1*50+2*0+3*0 | 50 | 0*50+1*50+2*0+3*0 | 50 | 50 |
| 190 | 0*20+1*0+2*0+3*80 | 240 | 0*20+1*0+2*0+3*80 | 240 | 0*20+1*0+2*0+3*80 | 240 | 240 | 0*10+1*90+2*0+3*0 | 90 | 0*10+1*90+2*0+3*0 | 90 | 0*10+1*90+2*0+3*0 | 90 | 90 |
| 191 | 0*0+1*0+2*100+3*0 | 200 | 0*0+1*0+2*100+3*0 | 200 | 0*0+1*0+2*100+3*0 | 200 | 200 | 0*50+1*0+2*50+3*0 | 100 | 0*0+1*90+2*10+3*0 | 110 | 0*40+1*0+2*60+3*0 | 120 | 110 |
| 192 | 0*0+1*0+2*100+3*0 | 200 | 0*0+1*0+2*100+3*0 | 200 | 0*0+1*0+2*100+3*0 | 200 | 200 | 0*10+1*0+2*90+3*0 | 180 | 0*10+1*0+2*90+3*0 | 180 | 0*10+1*0+2*90+3*0 | 180 | 180 |
| 193 | 0*50+1*0+2*0+3*50 | 150 | 0*50+1*0+2*0+3*50 | 150 | 0*50+1*0+2*0+3*50 | 150 | 150 | 0*50+1*0+2*0+3*50 | 150 | 0*50+1*0+2*0+3*50 | 150 | 0*50+1*0+2*0+3*50 | 150 | 150 |
| 194 | 0*30+1*0+2*0+3*70 | 210 | 0*0+1*40+2*0+3*60 | 220 | 0*20+1*0+2*10+3*70 | 230 | 220 | 0*0+1*40+2*0+3*60 | 220 | 0*0+1*40+2*0+3*60 | 220 | 0*0+1*40+2*0+3*60 | 220 | 220 |
| 195 | 0*50+1*0+2*0+3*50 | 150 | 0*50+1*0+2*0+3*50 | 150 | 0*50+1*0+2*0+3*50 | 150 | 150 | 0*30+1*70+2*0+3*0 | 70 | 0*30+1*70+2*0+3*0 | 70 | 0*30+1*70+2*0+3*0 | 70 | 70 |
| 196 | 0*0+1*40+2*0+3*60 | 220 | 0*0+1*40+2*0+3*60 | 220 | 0*0+1*40+2*0+3*60 | 220 | 220 | 0*20+1*0+2*0+3*80 | 240 | 0*20+1*0+2*0+3*80 | 240 | 0*20+1*0+2*0+3*80 | 240 | 240 |
| 197 | 0*20+1*0+2*0+3*80 | 240 | 0*20+1*0+2*0+3*80 | 240 | 0*20+1*0+2*0+3*80 | 240 | 240 | 0*20+1*0+2*80+3*0 | 160 | 0*20+1*0+2*80+3*0 | 160 | 0*20+1*0+2*80+3*0 | 160 | 160 |
| 198 | 0*50+1*0+2*0+3*50 | 150 | 0*50+1*0+2*0+3*50 | 150 | 0*50+1*0+2*0+3*50 | 150 | 150 | 0*50+1*0+2*50+3*0 | 100 | 0*50+1*0+2*50+3*0 | 100 | 0*50+1*0+2*50+3*0 | 100 | 100 |
| 199 | 0*0+1*40+2*0+3*60 | 220 | 0*20+1*0+2*0+3*80 | 240 | 0*0+1*20+2*0+3*80 | 260 | 240 | 0*30+1*0+2*70+3*0 | 140 | 0*0+1*50+2*50+3*0 | 150 | 0*0+1*10+2*90+3*0 | 190 | 160 |
| 200 | 0*20+1*0+2*80+3*0 | 160 | 0*20+1*0+2*80+3*0 | 160 | 0*20+1*0+2*80+3*0 | 160 | 160 | 0*40+1*0+2*60+3*0 | 120 | 0*40+1*0+2*60+3*0 | 120 | 0*40+1*0+2*60+3*0 | 120 | 120 |
| 201 | 0*0+1*30+2*70+3*0 | 170 | 0*0+1*30+2*70+3*0 | 170 | 0*0+1*30+2*70+3*0 | 170 | 170 | 0*30+1*0+2*0+3*70 | 210 | 0*30+1*0+2*0+3*70 | 210 | 0*30+1*0+2*0+3*70 | 210 | 210 |
| 202 | 0*50+1*0+2*50+3*0 | 100 | 0*50+1*0+2*50+3*0 | 100 | 0*50+1*0+2*50+3*0 | 100 | 100 | 0*0+1*10+2*90+3*0 | 190 | 0*0+1*10+2*90+3*0 | 190 | 0*0+1*10+2*90+3*0 | 190 | 190 |
| 203 | 0*40+1*0+2*0+3*60 | 180 | 0*40+1*0+2*0+3*60 | 180 | 0*40+1*0+2*0+3*60 | 180 | 180 | 0*10+1*0+2*90+3*0 | 180 | 0*10+1*0+2*90+3*0 | 180 | 0*10+1*0+2*90+3*0 | 180 | 180 |
| 204 | 0*40+1*0+2*0+3*60 | 180 | 0*40+1*0+2*0+3*60 | 180 | 0*40+1*0+2*0+3*60 | 180 | 180 | 0*10+1*90+2*0+3*0 | 90 | 0*10+1*90+2*0+3*0 | 90 | 0*10+1*90+2*0+3*0 | 90 | 90 |
| 205 | 0*0+1*40+2*0+3*60 | 220 | 0*0+1*40+2*0+3*60 | 220 | 0*0+1*40+2*0+3*60 | 220 | 220 | 0*10+1*0+2*90+3*0 | 180 | 0*10+1*0+2*90+3*0 | 180 | 0*10+1*0+2*90+3*0 | 180 | 180 |
| 206 | 0*20+1*0+2*10+3*70 | 230 | 0*0+1*40+2*0+3*60 | 220 | 0*20+1*0+2*0+3*80 | 240 | 230 | 0*0+1*90+2*10+3*0 | 110 | 0*40+1*0+2*60+3*0 | 120 | 0*20+1*0+2*80+3*0 | 160 | 130 |
| 207 | 0*10+1*10+2*0+3*80 | 250 | 0*10+1*10+2*0+3*80 | 250 | 0*10+1*10+2*0+3*80 | 250 | 250 | 0*20+1*0+2*80+3*0 | 160 | 0*0+1*30+2*70+3*0 | 170 | 0*10+1*0+2*90+3*0 | 180 | 170 |
| 208 | 0*20+1*0+2*0+3*80 | 240 | 0*20+1*0+2*0+3*80 | 240 | 0*20+1*0+2*0+3*80 | 240 | 240 | 0*0+1*90+2*10+3*0 | 110 | 0*40+1*0+2*60+3*0 | 120 | 0*20+1*0+2*80+3*0 | 160 | 130 |
| 209 | 0*30+1*0+2*0+3*70 | 210 | 0*0+1*40+2*0+3*60 | 220 | 0*20+1*0+2*10+3*70 | 230 | 220 | 0*50+1*0+2*50+3*0 | 100 | 0*50+1*0+2*50+3*0 | 100 | 0*50+1*0+2*50+3*0 | 100 | 100 |
| 210 | 0*0+1*0+2*100+3*0 | 200 | 0*20+1*0+2*10+3*70 | 230 | 0*0+1*20+2*0+3*80 | 260 | 230 | 0*50+1*0+2*0+3*50 | 150 | 0*50+1*0+2*0+3*50 | 150 | 0*50+1*0+2*0+3*50 | 150 | 150 |
| 211 | 0*40+1*0+2*0+3*60 | 180 | 0*40+1*0+2*0+3*60 | 180 | 0*40+1*0+2*0+3*60 | 180 | 180 | 0*50+1*0+2*0+3*50 | 150 | 0*50+1*0+2*0+3*50 | 150 | 0*50+1*0+2*0+3*50 | 150 | 150 |
| 212 | 0*50+1*50+2*0+3*0 | 50 | 0*50+1*50+2*0+3*0 | 50 | 0*50+1*50+2*0+3*0 | 50 | 50 | 0*10+1*0+2*90+3*0 | 180 | 0*10+1*0+2*90+3*0 | 180 | 0*10+1*0+2*90+3*0 | 180 | 180 |
| 213 | 0*30+1*0+2*70+3*0 | 140 | 0*50+1*0+2*0+3*50 | 150 | 0*20+1*0+2*80+3*0 | 160 | 150 | 0*0+1*70+2*30+3*0 | 130 | 0*30+1*0+2*70+3*0 | 140 | 0*0+1*50+2*50+3*0 | 150 | 140 |
| 214 | 0*50+1*0+2*0+3*50 | 150 | 0*50+1*0+2*0+3*50 | 150 | 0*50+1*0+2*0+3*50 | 150 | 150 | 0*0+1*40+2*0+3*60 | 220 | 0*0+1*0+2*70+3*30 | 230 | 0*20+1*0+2*0+3*80 | 240 | 230 |
| 215 | 0*0+1*10+2*0+3*90 | 280 | 0*0+1*0+2*10+3*90 | 290 | 0*0+1*0+2*0+3*100 | 300 | 290 | 0*20+1*80+2*0+3*0 | 80 | 0*10+1*90+2*0+3*0 | 90 | 0*0+1*100+2*0+3*0 | 100 | 90 |
| 216 | 0*10+1*10+2*0+3*80 | 250 | 0*0+1*20+2*0+3*80 | 260 | 0*10+1*0+2*0+3*90 | 270 | 260 | 0*50+1*0+2*50+3*0 | 100 | 0*0+1*90+2*10+3*0 | 110 | 0*40+1*0+2*60+3*0 | 120 | 110 |
| 217 | 0*40+1*10+2*50+3*0 | 110 | 0*40+1*10+2*50+3*0 | 110 | 0*40+1*10+2*50+3*0 | 110 | 110 | 0*10+1*0+2*90+3*0 | 180 | 0*10+1*0+2*90+3*0 | 180 | 0*10+1*0+2*90+3*0 | 180 | 180 |
| 218 | 0*50+1*0+2*0+3*50 | 150 | 0*50+1*0+2*0+3*50 | 150 | 0*50+1*0+2*0+3*50 | 150 | 150 | 0*40+1*0+2*60+3*0 | 120 | 0*0+1*70+2*30+3*0 | 130 | 0*30+1*0+2*70+3*0 | 140 | 130 |
| 219 | 0*70+1*30+2*0+3*0 | 30 | 0*70+1*30+2*0+3*0 | 30 | 0*70+1*30+2*0+3*0 | 30 | 30 | 0*40+1*0+2*60+3*0 | 120 | 0*40+1*0+2*60+3*0 | 120 | 0*40+1*0+2*60+3*0 | 120 | 120 |
| 220 | 0*10+1*0+2*90+3*0 | 180 | 0*0+1*10+2*90+3*0 | 190 | 0*0+1*0+2*100+3*0 | 200 | 190 | 0*20+1*0+2*80+3*0 | 160 | 0*0+1*30+2*70+3*0 | 170 | 0*10+1*0+2*90+3*0 | 180 | 170 |
| 221 | 0*10+1*0+2*90+3*0 | 180 | 0*10+1*0+2*90+3*0 | 180 | 0*10+1*0+2*90+3*0 | 180 | 180 | 0*50+1*0+2*50+3*0 | 100 | 0*50+1*0+2*50+3*0 | 100 | 0*50+1*0+2*50+3*0 | 100 | 100 |
| 222 | 0*20+1*0+2*80+3*0 | 160 | 0*0+1*30+2*70+3*0 | 170 | 0*40+1*0+2*0+3*60 | 180 | 170 | 0*0+1*70+2*30+3*0 | 130 | 0*30+1*0+2*70+3*0 | 140 | 0*0+1*50+2*50+3*0 | 150 | 140 |
| 223 | 0*40+1*0+2*0+3*60 | 180 | 0*0+1*10+2*90+3*0 | 190 | 0*0+1*0+2*100+3*0 | 200 | 190 | 0*0+1*30+2*70+3*0 | 170 | 0*0+1*30+2*70+3*0 | 170 | 0*0+1*30+2*70+3*0 | 170 | 170 |
| 224 | 0*10+1*10+2*0+3*80 | 250 | 0*10+1*10+2*0+3*80 | 250 | 0*10+1*10+2*0+3*80 | 250 | 250 | 0*10+1*0+2*90+3*0 | 180 | 0*0+1*10+2*90+3*0 | 190 | 0*0+1*0+2*100+3*0 | 200 | 190 |
| 225 | 0*0+1*30+2*70+3*0 | 170 | 0*0+1*30+2*70+3*0 | 170 | 0*0+1*30+2*70+3*0 | 170 | 170 | 0*0+1*30+2*70+3*0 | 170 | 0*0+1*30+2*70+3*0 | 170 | 0*0+1*30+2*70+3*0 | 170 | 170 |
| 226 | 0*50+1*0+2*0+3*50 | 150 | 0*20+1*0+2*80+3*0 | 160 | 0*0+1*30+2*70+3*0 | 170 | 160 | 0*30+1*0+2*70+3*0 | 140 | 0*0+1*50+2*50+3*0 | 150 | 0*20+1*0+2*80+3*0 | 160 | 150 |
| 227 | 0*30+1*70+2*0+3*0 | 70 | 0*30+1*70+2*0+3*0 | 70 | 0*30+1*70+2*0+3*0 | 70 | 70 | 0*20+1*0+2*80+3*0 | 160 | 0*0+1*30+2*70+3*0 | 170 | 0*10+1*0+2*90+3*0 | 180 | 170 |
| 228 | 0*0+1*70+2*30+3*0 | 130 | 0*0+1*70+2*30+3*0 | 130 | 0*0+1*70+2*30+3*0 | 130 | 130 | 0*30+1*0+2*70+3*0 | 140 | 0*30+1*0+2*70+3*0 | 140 | 0*30+1*0+2*70+3*0 | 140 | 140 |
| 229 | 0*60+1*0+2*40+3*0 | 80 | 0*70+1*0+2*0+3*30 | 90 | 0*50+1*0+2*50+3*0 | 100 | 90 | 0*0+1*70+2*30+3*0 | 130 | 0*30+1*0+2*70+3*0 | 140 | 0*0+1*50+2*50+3*0 | 150 | 140 |
| 230 | 0*20+1*0+2*80+3*0 | 160 | 0*0+1*30+2*70+3*0 | 170 | 0*10+1*0+2*90+3*0 | 180 | 170 | 0*0+1*0+2*100+3*0 | 200 | 0*30+1*0+2*0+3*70 | 210 | 0*0+1*40+2*0+3*60 | 220 | 210 |
| 231 | 0*0+1*100+2*0+3*0 | 100 | 0*40+1*10+2*50+3*0 | 110 | 0*40+1*0+2*60+3*0 | 120 | 110 | 0*40+1*0+2*60+3*0 | 120 | 0*40+1*0+2*60+3*0 | 120 | 0*40+1*0+2*60+3*0 | 120 | 120 |
| 232 | 0*50+1*50+2*0+3*0 | 50 | 0*50+1*50+2*0+3*0 | 50 | 0*50+1*50+2*0+3*0 | 50 | 50 | 0*10+1*0+2*90+3*0 | 180 | 0*10+1*0+2*90+3*0 | 180 | 0*10+1*0+2*90+3*0 | 180 | 180 |
| 233 | 0*10+1*0+2*90+3*0 | 180 | 0*0+1*10+2*90+3*0 | 190 | 0*0+1*0+2*100+3*0 | 200 | 190 | 0*0+1*100+2*0+3*0 | 100 | 0*0+1*100+2*0+3*0 | 100 | 0*0+1*100+2*0+3*0 | 100 | 100 |
| 234 | 0*0+1*40+2*0+3*60 | 220 | 0*0+1*40+2*0+3*60 | 220 | 0*0+1*40+2*0+3*60 | 220 | 220 | 0*70+1*30+2*0+3*0 | 30 | 0*70+1*30+2*0+3*0 | 30 | 0*70+1*30+2*0+3*0 | 30 | 30 |
| 235 | 0*50+1*0+2*0+3*50 | 150 | 0*50+1*0+2*0+3*50 | 150 | 0*50+1*0+2*0+3*50 | 150 | 150 | 0*0+1*0+2*100+3*0 | 200 | 0*0+1*50+2*50+3*0 | 150 | 0*20+1*0+2*80+3*0 | 160 | 170 |
| 236 | 0*30+1*0+2*70+3*0 | 140 | 0*30+1*0+2*70+3*0 | 140 | 0*30+1*0+2*70+3*0 | 140 | 140 | 0*10+1*0+2*90+3*0 | 180 | 0*10+1*0+2*90+3*0 | 180 | 0*10+1*0+2*90+3*0 | 180 | 180 |
| 237 | 0*20+1*0+2*80+3*0 | 160 | 0*0+1*30+2*70+3*0 | 170 | 0*10+1*0+2*90+3*0 | 180 | 170 | 0*50+1*0+2*50+3*0 | 100 | 0*0+1*90+2*10+3*0 | 110 | 0*40+1*0+2*60+3*0 | 120 | 110 |
| 238 | 0*0+1*30+2*70+3*0 | 170 | 0*0+1*30+2*70+3*0 | 170 | 0*0+1*30+2*70+3*0 | 170 | 170 | 0*0+1*50+2*50+3*0 | 150 | 0*20+1*0+2*80+3*0 | 160 | 0*0+1*30+2*70+3*0 | 170 | 160 |
| 239 | 0*20+1*0+2*80+3*0 | 160 | 0*0+1*30+2*70+3*0 | 170 | 0*10+1*0+2*90+3*0 | 180 | 170 | 0*0+1*0+2*50+3*50 | 250 | 0*0+1*0+2*50+3*50 | 250 | 0*0+1*0+2*50+3*50 | 250 | 250 |
| 240 | 0*0+1*100+2*0+3*0 | 100 | 0*0+1*100+2*0+3*0 | 100 | 0*0+1*100+2*0+3*0 | 100 | 100 | 0*0+1*0+2*100+3*0 | 200 | 0*30+1*0+2*0+3*70 | 210 | 0*0+1*40+2*0+3*60 | 220 | 210 |
| 241 | 0*0+1*100+2*0+3*0 | 100 | 0*40+1*0+2*60+3*0 | 120 | 0*0+1*90+2*10+3*0 | 110 | 110 | 0*10+1*0+2*90+3*0 | 180 | 0*10+1*0+2*90+3*0 | 180 | 0*10+1*0+2*90+3*0 | 180 | 180 |
| 242 | 0*50+1*0+2*0+3*50 | 150 | 0*20+1*0+2*80+3*0 | 160 | 0*0+1*0+2*100+3*0 | 200 | 170 | 0*10+1*90+2*0+3*0 | 90 | 0*10+1*90+2*0+3*0 | 90 | 0*10+1*90+2*0+3*0 | 90 | 90 |
| 243 | 0*50+1*0+2*50+3*0 | 100 | 0*50+1*0+2*50+3*0 | 100 | 0*50+1*0+2*50+3*0 | 100 | 100 | 0*40+1*60+2*0+3*0 | 60 | 0*30+1*70+2*0+3*0 | 70 | 0*20+1*80+2*0+3*0 | 80 | 70 |
| 244 | 0*10+1*0+2*90+3*0 | 180 | 0*0+1*0+2*100+3*0 | 200 | 0*0+1*10+2*90+3*0 | 190 | 190 | 0*0+1*10+2*90+3*0 | 190 | 0*0+1*10+2*90+3*0 | 190 | 0*0+1*10+2*90+3*0 | 190 | 190 |
| 245 | 0*0+1*30+2*70+3*0 | 170 | 0*10+1*0+2*90+3*0 | 180 | 0*0+1*40+2*0+3*60 | 220 | 190 | 0*10+1*0+2*90+3*0 | 180 | 0*10+1*0+2*90+3*0 | 180 | 0*10+1*0+2*90+3*0 | 180 | 180 |
| 246 | 0*10+1*0+2*90+3*0 | 180 | 0*10+1*0+2*90+3*0 | 180 | 0*10+1*0+2*90+3*0 | 180 | 180 | 0*20+1*0+2*80+3*0 | 160 | 0*0+1*30+2*70+3*0 | 170 | 0*10+1*0+2*90+3*0 | 180 | 170 |
| 247 | 0*0+1*30+2*70+3*0 | 170 | 0*0+1*30+2*70+3*0 | 170 | 0*0+1*0+2*100+3*0 | 200 | 180 | 0*0+1*40+2*0+3*60 | 220 | 0*0+1*0+2*70+3*30 | 230 | 0*20+1*0+2*0+3*80 | 240 | 230 |
| 248 | 0*20+1*0+2*0+3*80 | 240 | 0*20+1*0+2*0+3*80 | 240 | 0*20+1*0+2*0+3*80 | 240 | 240 | 0*20+1*0+2*0+3*80 | 240 | 0*20+1*0+2*0+3*80 | 240 | 0*20+1*0+2*0+3*80 | 240 | 240 |
| 249 | 0*50+1*50+2*0+3*0 | 50 | 0*50+1*50+2*0+3*0 | 50 | 0*50+1*50+2*0+3*0 | 50 | 50 | 0*0+1*100+2*0+3*0 | 100 | 0*0+1*100+2*0+3*0 | 100 | 0*0+1*100+2*0+3*0 | 100 | 100 |
| 250 | 0*0+1*0+2*100+3*0 | 200 | 0*0+1*0+2*100+3*0 | 200 | 0*0+1*0+2*100+3*0 | 200 | 200 | 0*20+1*80+2*0+3*0 | 80 | 0*10+1*90+2*0+3*0 | 90 | 0*0+1*100+2*0+3*0 | 100 | 90 |
| 251 | 0*10+1*0+2*90+3*0 | 180 | 0*0+1*10+2*90+3*0 | 190 | 0*0+1*0+2*100+3*0 | 200 | 190 | 0*10+1*0+2*90+3*0 | 180 | 0*10+1*0+2*90+3*0 | 180 | 0*10+1*0+2*90+3*0 | 180 | 180 |
| 252 | 0*20+1*0+2*0+3*80 | 240 | 0*20+1*0+2*0+3*80 | 240 | 0*20+1*0+2*0+3*80 | 240 | 240 | 0*0+1*100+2*0+3*0 | 100 | 0*0+1*100+2*0+3*0 | 100 | 0*0+1*100+2*0+3*0 | 100 | 100 |
| 253 | 0*20+1*0+2*80+3*0 | 160 | 0*20+1*0+2*80+3*0 | 160 | 0*20+1*0+2*80+3*0 | 160 | 160 | 0*10+1*90+2*0+3*0 | 90 | 0*10+1*90+2*0+3*0 | 90 | 0*10+1*90+2*0+3*0 | 90 | 90 |
| 254 | 0*30+1*0+2*0+3*70 | 210 | 0*0+1*40+2*0+3*60 | 220 | 0*10+1*0+2*40+3*50 | 230 | 220 | 0*10+1*0+2*90+3*0 | 180 | 0*0+1*10+2*90+3*0 | 190 | 0*0+1*0+2*100+3*0 | 200 | 190 |
| 255 | 0*0+1*0+2*50+3*50 | 250 | 0*0+1*0+2*50+3*50 | 250 | 0*0+1*0+2*50+3*50 | 250 | 250 | 0*10+1*0+2*90+3*0 | 180 | 0*0+1*10+2*90+3*0 | 190 | 0*0+1*0+2*100+3*0 | 200 | 190 |
| 256 | 0*0+1*30+2*70+3*0 | 170 | 0*0+1*30+2*70+3*0 | 170 | 0*0+1*30+2*70+3*0 | 170 | 170 | 0*0+1*30+2*70+3*0 | 170 | 0*0+1*30+2*70+3*0 | 170 | 0*0+1*30+2*70+3*0 | 170 | 170 |
| 257 | 0*10+1*0+2*0+3*90 | 270 | 0*0+1*10+2*0+3*90 | 280 | 0*0+1*0+2*10+3*90 | 290 | 280 | 0*50+1*50+2*0+3*0 | 50 | 0*50+1*50+2*0+3*0 | 50 | 0*50+1*50+2*0+3*0 | 50 | 50 |
| 258 | 0*10+1*0+2*0+3*90 | 270 | 0*0+1*10+2*0+3*90 | 280 | 0*0+1*0+2*10+3*90 | 290 | 280 | 0*10+1*0+2*90+3*0 | 180 | 0*10+1*0+2*90+3*0 | 180 | 0*10+1*0+2*90+3*0 | 180 | 180 |
| 259 | 0*0+1*0+2*0+3*100 | 300 | 0*10+1*0+2*0+3*90 | 270 | 0*0+1*0+2*0+3*100 | 300 | 290 | 0*30+1*70+2*0+3*0 | 70 | 0*20+1*80+2*0+3*0 | 80 | 0*40+1*0+2*60+3*0 | 120 | 90 |
| 260 | 0*10+1*0+2*40+3*50 | 230 | 0*10+1*0+2*40+3*50 | 230 | 0*10+1*0+2*40+3*50 | 230 | 230 | 0*30+1*70+2*0+3*0 | 70 | 0*30+1*70+2*0+3*0 | 70 | 0*30+1*70+2*0+3*0 | 70 | 70 |
| 261 | 0*0+1*40+2*0+3*60 | 220 | 0*10+1*0+2*40+3*50 | 230 | 0*20+1*0+2*0+3*80 | 240 | 230 | 0*40+1*0+2*60+3*0 | 120 | 0*40+1*0+2*60+3*0 | 120 | 0*40+1*0+2*60+3*0 | 120 | 120 |
| 262 | 0*50+1*50+2*0+3*0 | 50 | 0*50+1*50+2*0+3*0 | 50 | 0*50+1*50+2*0+3*0 | 50 | 50 | 0*40+1*0+2*60+3*0 | 120 | 0*0+1*70+2*30+3*0 | 130 | 0*30+1*0+2*70+3*0 | 140 | 130 |
| 263 | 0*20+1*0+2*0+3*80 | 240 | 0*20+1*0+2*0+3*80 | 240 | 0*20+1*0+2*0+3*80 | 240 | 240 | 0*0+1*40+2*0+3*60 | 220 | 0*0+1*40+2*0+3*60 | 220 | 0*0+1*40+2*0+3*60 | 220 | 220 |
| 264 | 0*0+1*40+2*0+3*60 | 220 | 0*20+1*0+2*10+3*70 | 230 | 0*20+1*0+2*0+3*80 | 240 | 230 | 0*0+1*70+2*30+3*0 | 130 | 0*30+1*0+2*70+3*0 | 140 | 0*0+1*50+2*50+3*0 | 150 | 140 |
| 265 | 0*0+1*0+2*50+3*50 | 250 | 0*0+1*0+2*50+3*50 | 250 | 0*0+1*0+2*50+3*50 | 250 | 250 | 0*40+1*60+2*0+3*0 | 60 | 0*30+1*70+2*0+3*0 | 70 | 0*20+1*80+2*0+3*0 | 80 | 70 |
| 266 | 0*0+1*0+2*50+3*50 | 250 | 0*0+1*0+2*50+3*50 | 250 | 0*0+1*10+2*0+3*90 | 280 | 260 | 0*20+1*0+2*80+3*0 | 160 | 0*20+1*0+2*80+3*0 | 160 | 0*20+1*0+2*80+3*0 | 160 | 160 |
| 267 | 0*0+1*40+2*0+3*60 | 220 | 0*10+1*20+2*0+3*70 | 230 | 0*20+1*0+2*0+3*80 | 240 | 230 | 0*0+1*0+2*50+3*50 | 250 | 0*0+1*20+2*0+3*80 | 260 | 0*10+1*0+2*0+3*90 | 270 | 260 |
| 268 | 0*0+1*40+2*0+3*60 | 220 | 0*0+1*40+2*0+3*60 | 220 | 0*0+1*40+2*0+3*60 | 220 | 220 | 0*0+1*100+2*0+3*0 | 100 | 0*0+1*100+2*0+3*0 | 100 | 0*0+1*100+2*0+3*0 | 100 | 100 |
| 269 | 0*30+1*0+2*0+3*70 | 210 | 0*0+1*40+2*0+3*60 | 220 | 0*10+1*20+2*0+3*70 | 230 | 220 | 0*0+1*0+2*100+3*0 | 200 | 0*0+1*0+2*100+3*0 | 200 | 0*0+1*0+2*100+3*0 | 200 | 200 |
| 270 | 0*60+1*0+2*0+3*40 | 120 | 0*60+1*0+2*0+3*40 | 120 | 0*60+1*0+2*0+3*40 | 120 | 120 | 0*0+1*70+2*30+3*0 | 130 | 0*0+1*70+2*30+3*0 | 130 | 0*0+1*70+2*30+3*0 | 130 | 130 |
| 271 | 0*0+1*40+2*0+3*60 | 220 | 0*0+1*40+2*0+3*60 | 220 | 0*0+1*0+2*50+3*50 | 250 | 230 | 0*0+1*100+2*0+3*0 | 100 | 0*0+1*100+2*0+3*0 | 100 | 0*0+1*100+2*0+3*0 | 100 | 100 |
| 272 | 0*60+1*0+2*40+3*0 | 80 | 0*60+1*0+2*40+3*0 | 80 | 0*60+1*0+2*40+3*0 | 80 | 80 | 0*70+1*30+2*0+3*0 | 30 | 0*70+1*30+2*0+3*0 | 30 | 0*70+1*30+2*0+3*0 | 30 | 30 |
| 273 | 0*0+1*50+2*0+3*50 | 200 | 0*0+1*50+2*0+3*50 | 200 | 0*0+1*50+2*0+3*50 | 200 | 200 | 0*40+1*0+2*60+3*0 | 120 | 0*0+1*70+2*30+3*0 | 130 | 0*30+1*0+2*70+3*0 | 140 | 130 |
| 274 | 0*30+1*0+2*0+3*70 | 210 | 0*0+1*40+2*0+3*60 | 220 | 0*10+1*20+2*0+3*70 | 230 | 220 | 0*0+1*0+2*100+3*0 | 200 | 0*0+1*50+2*50+3*0 | 150 | 0*20+1*0+2*80+3*0 | 160 | 170 |
| 275 | 0*0+1*0+2*50+3*50 | 250 | 0*0+1*0+2*50+3*50 | 250 | 0*0+1*0+2*50+3*50 | 250 | 250 | 0*10+1*90+2*0+3*0 | 90 | 0*10+1*90+2*0+3*0 | 90 | 0*10+1*90+2*0+3*0 | 90 | 90 |
| 276 | 0*10+1*0+2*90+3*0 | 180 | 0*0+1*10+2*90+3*0 | 190 | 0*0+1*0+2*100+3*0 | 200 | 190 | 0*40+1*0+2*60+3*0 | 120 | 0*0+1*70+2*30+3*0 | 130 | 0*30+1*0+2*70+3*0 | 140 | 130 |
| 277 | 0*0+1*0+2*100+3*0 | 200 | 0*0+1*0+2*100+3*0 | 200 | 0*0+1*0+2*100+3*0 | 200 | 200 | 0*10+1*0+2*90+3*0 | 180 | 0*10+1*0+2*90+3*0 | 180 | 0*10+1*0+2*90+3*0 | 180 | 180 |
| 278 | 0*0+1*50+2*0+3*50 | 200 | 0*30+1*0+2*0+3*70 | 210 | 0*0+1*0+2*50+3*50 | 250 | 220 | 0*0+1*100+2*0+3*0 | 100 | 0*0+1*100+2*0+3*0 | 100 | 0*0+1*100+2*0+3*0 | 100 | 100 |
| 279 | 0*0+1*50+2*0+3*50 | 200 | 0*0+1*50+2*0+3*50 | 200 | 0*0+1*50+2*0+3*50 | 200 | 200 | 0*0+1*100+2*0+3*0 | 100 | 0*10+1*90+2*0+3*0 | 90 | 0*20+1*80+2*0+3*0 | 80 | 90 |
| 280 | 0*30+1*0+2*0+3*70 | 210 | 0*30+1*0+2*0+3*70 | 210 | 0*20+1*0+2*0+3*80 | 240 | 220 | 0*0+1*100+2*0+3*0 | 100 | 0*0+1*100+2*0+3*0 | 100 | 0*0+1*70+2*30+3*0 | 130 | 110 |
| 281 | 0*10+1*0+2*40+3*50 | 230 | 0*10+1*0+2*40+3*50 | 230 | 0*10+1*0+2*40+3*50 | 230 | 230 | 0*20+1*0+2*80+3*0 | 160 | 0*0+1*30+2*70+3*0 | 170 | 0*10+1*0+2*90+3*0 | 180 | 170 |
| 282 | 0*0+1*90+2*10+3*0 | 110 | 0*0+1*70+2*30+3*0 | 130 | 0*50+1*0+2*0+3*50 | 150 | 130 | 0*0+1*90+2*10+3*0 | 110 | 0*0+1*70+2*30+3*0 | 130 | 0*40+1*0+2*60+3*0 | 120 | 120 |
| 283 | 0*0+1*40+2*0+3*60 | 220 | 0*0+1*40+2*0+3*60 | 220 | 0*0+1*40+2*0+3*60 | 220 | 220 | 0*40+1*60+2*0+3*0 | 60 | 0*20+1*80+2*0+3*0 | 80 | 0*0+1*100+2*0+3*0 | 100 | 80 |
| 284 | 0*0+1*50+2*0+3*50 | 200 | 0*30+1*0+2*0+3*70 | 210 | 0*0+1*0+2*50+3*50 | 250 | 220 | 0*0+1*100+2*0+3*0 | 100 | 0*0+1*100+2*0+3*0 | 100 | 0*0+1*100+2*0+3*0 | 100 | 100 |
| 285 | 0*0+1*10+2*0+3*90 | 280 | 0*0+1*0+2*20+3*80 | 280 | 0*0+1*0+2*20+3*80 | 280 | 280 | 0*0+1*30+2*70+3*0 | 170 | 0*10+1*0+2*90+3*0 | 180 | 0*0+1*10+2*90+3*0 | 190 | 180 |
| 286 | 0*0+1*0+2*10+3*90 | 290 | 0*0+1*0+2*20+3*80 | 280 | 0*10+1*0+2*0+3*90 | 270 | 280 | 0*0+1*90+2*10+3*0 | 110 | 0*0+1*90+2*10+3*0 | 110 | 0*0+1*90+2*10+3*0 | 110 | 110 |
| 287 | 0*10+1*0+2*90+3*0 | 180 | 0*10+1*0+2*90+3*0 | 180 | 0*10+1*0+2*90+3*0 | 180 | 180 | 0*0+1*90+2*10+3*0 | 110 | 0*0+1*70+2*30+3*0 | 130 | 0*0+1*50+2*50+3*0 | 150 | 130 |
| 288 | 0*50+1*50+2*0+3*0 | 50 | 0*40+1*60+2*0+3*0 | 60 | 0*30+1*70+2*0+3*0 | 70 | 60 | 0*0+1*100+2*0+3*0 | 100 | 0*0+1*100+2*0+3*0 | 100 | 0*0+1*100+2*0+3*0 | 100 | 100 |
| 289 | 0*30+1*0+2*0+3*70 | 210 | 0*30+1*0+2*0+3*70 | 210 | 0*30+1*0+2*0+3*70 | 210 | 210 | 0*0+1*100+2*0+3*0 | 100 | 0*0+1*100+2*0+3*0 | 100 | 0*0+1*100+2*0+3*0 | 100 | 100 |
| 290 | 0*0+1*30+2*70+3*0 | 170 | 0*0+1*10+2*90+3*0 | 190 | 0*30+1*0+2*0+3*70 | 210 | 190 | 0*20+1*0+2*80+3*0 | 160 | 0*20+1*0+2*80+3*0 | 160 | 0*0+1*10+2*90+3*0 | 190 | 170 |
| 291 | 0*10+1*0+2*90+3*0 | 180 | 0*10+1*0+2*90+3*0 | 180 | 0*10+1*0+2*90+3*0 | 180 | 180 | 0*30+1*70+2*0+3*0 | 70 | 0*20+1*80+2*0+3*0 | 80 | 0*10+1*90+2*0+3*0 | 90 | 80 |
| 292 | 0*20+1*0+2*10+3*70 | 230 | 0*20+1*0+2*10+3*70 | 230 | 0*0+1*20+2*0+3*80 | 260 | 240 | 0*0+1*0+2*0+3*0 | 0 | 0*0+1*0+2*0+3*0 | 0 | 0*0+1*0+2*0+3*0 | 0 | 0 |
| 293 | 0*20+1*0+2*80+3*0 | 160 | 0*0+1*30+2*70+3*0 | 170 | 0*10+1*0+2*90+3*0 | 180 | 170 | 0*60+1*40+2*0+3*0 | 40 | 0*60+1*40+2*0+3*0 | 40 | 0*60+1*40+2*0+3*0 | 40 | 40 |
| 294 | 0*10+1*20+2*0+3*70 | 230 | 0*10+1*20+2*0+3*70 | 230 | 0*10+1*20+2*0+3*70 | 230 | 230 | 0*50+1*50+2*0+3*0 | 50 | 0*50+1*50+2*0+3*0 | 50 | 0*50+1*50+2*0+3*0 | 50 | 50 |
| 295 | 0*10+1*0+2*90+3*0 | 180 | 0*0+1*10+2*90+3*0 | 190 | 0*0+1*0+2*100+3*0 | 200 | 190 | 0*80+1*20+2*0+3*0 | 20 | 0*80+1*20+2*0+3*0 | 20 | 0*80+1*20+2*0+3*0 | 20 | 20 |
| 296 | 0*0+1*0+2*20+3*80 | 280 | 0*0+1*0+2*20+3*80 | 280 | 0*0+1*0+2*20+3*80 | 280 | 280 | 0*50+1*50+2*0+3*0 | 50 | 0*40+1*60+2*0+3*0 | 60 | 0*30+1*70+2*0+3*0 | 70 | 60 |
| 297 | 0*20+1*0+2*0+3*80 | 240 | 0*0+1*0+2*50+3*50 | 250 | 0*0+1*20+2*0+3*80 | 260 | 250 | 0*0+1*100+2*0+3*0 | 100 | 0*0+1*100+2*0+3*0 | 100 | 0*0+1*100+2*0+3*0 | 100 | 100 |
| 298 | 0*20+1*0+2*0+3*80 | 240 | 0*20+1*0+2*0+3*80 | 240 | 0*20+1*0+2*0+3*80 | 240 | 240 | 0*60+1*40+2*0+3*0 | 40 | 0*60+1*40+2*0+3*0 | 40 | 0*60+1*40+2*0+3*0 | 40 | 40 |
| 299 | 0*0+1*40+2*0+3*60 | 220 | 0*0+1*40+2*0+3*60 | 220 | 0*0+1*40+2*0+3*60 | 220 | 220 | 0*40+1*60+2*0+3*0 | 60 | 0*20+1*80+2*0+3*0 | 80 | 0*0+1*100+2*0+3*0 | 100 | 80 |
| 300 | 0*10+1*0+2*90+3*0 | 180 | 0*30+1*0+2*0+3*70 | 210 | 0*10+1*0+2*90+3*0 | 180 | 190 | 0*50+1*50+2*0+3*0 | 50 | 0*20+1*80+2*0+3*0 | 80 | 0*20+1*80+2*0+3*0 | 80 | 70 |

*p* value <0.001(tumor vs normal)
